# Phagocytosed Photoreceptor Outer Segment Particles Within the Retinal Pigment Epithelium Show Diurnal Rhythmicity and Variation Between Cone Subtypes in Larval Zebrafish

**DOI:** 10.1096/fj.202500211R

**Published:** 2025-07-24

**Authors:** Jenni Partinen, Noora Emilia Nevala, Sanni Erämies, Teemu Olavi Ihalainen, Soile Nymark

**Affiliations:** ^1^ Faculty of Medicine and Health Technology Tampere University Tampere Finland; ^2^ Centre for Genomic Regulation Barcelona Catalonia Spain

**Keywords:** circadian clocks, cone photoreceptor, phagocytosis, retinal photoreceptor outer segment, retinal pigment epithelium, rhythmicity, zebrafish

## Abstract

Phagocytosis of retinal rod and cone outer segment (OS) tips by the retinal pigment epithelium (RPE) occurs daily to prevent the accumulation of harmful compounds in the photoreceptors. Rhythmic bursts seen as increased numbers of phagocytosed OS particles in the RPE are known to appear once or twice a day depending on the animal species. Yet, the variation of this rhythmicity between the distinct photoreceptor types is not well understood. We used zebrafish to compare the phagosome numbers and their daily rhythms between the different cone subtypes. We immunolabeled the different cone opsins from the histological sections of the eyes of zebrafish larvae that were collected at seven different time points throughout a 24 h circadian cycle. Internalized OS particles were then quantified using confocal microscopy and image analysis. Interestingly, the results revealed the presence of OS particles of all cone subtypes in the RPE throughout the day in larval zebrafish. However, we observed a significant increase in the phagosome numbers from UV and blue cones at two time points, whereas the number of green cone OS particles was more constant, probably reflecting their more immature developmental stage. We also investigated whether the rhythmicity is regulated by external light by keeping the larvae in constant darkness before sample preparation. We found that the complete darkness condition diminished the phagosome numbers of all cone subtypes and abolished the daytime peaks in the UV and blue cones, indicating that the rhythmicity is strongly affected by the external light in the larval zebrafish. Our findings provide new understanding on the rhythmicity of cone OS phagocytosis and its regulation.

## Graphical abstract

Retinal pigment epithelium (RPE) in the eye internalizes and digests aged outer segment (OS) tips by phagocytosis. This is a rhythmic process controlled by ambient light and circadian regulation. Here, we show that all cone subtypes in larval zebrafish are phagocytosed, but daily rhythmic variations in the OS particle numbers were observed with UV and blue cone OSs. Elimination of external light diminished the phenomenon indicating that the rhythmicity is substantially affected by the light–dark transitions in developing zebrafish.

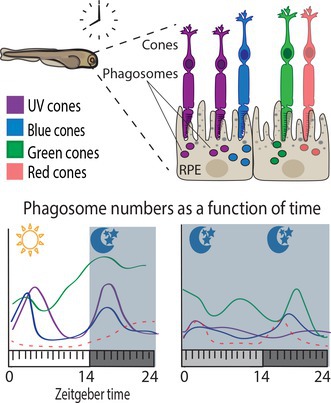

**Abbreviations**
dpf: days post fertilization
EM: electron microscopy
IS: inner segment
ONL: outer nuclear layer
OPL: outer plexiform layer
OS: outer segment
RPE: retinal pigment epithelium
ZT: Zeitgeber time

## 1 Introduction

Retinal photoreceptors, rods and cones, convert light information into electrical signals that are eventually conveyed to the brain. Rods function primarily at low‐light levels, whereas in most species, cones are active in bright light and are responsible for high spatial acuity and color vision. The first molecular players in visual perception are the light‐absorbing opsin proteins that are stored in the outer segments (OSs) of the photoreceptors and are specific to each photoreceptor type [1]. Retaining their light sensitivity and overall wellbeing depends on the retinal pigment epithelium (RPE) that forms a tight interlocked structure with the photoreceptor OSs via the apical microvilli [2]. The OSs of retinal photoreceptors are continuously exposed to high‐energy light from the environment, and thus, they are prone to light‐induced damages. To prevent excessive accumulation of harmful photo‐oxidative molecular compounds in the photoreceptors, the most distal and aged tips of the OSs are taken up by the RPE as small OS particles while new OS membrane is continuously generated at the OS base [3, 4, 5, 6]. This process has been traditionally called phagocytosis performed by the RPE. Yet, evidence from recent studies has suggested that the uptake process is, instead, more trogocytosis‐like, as RPE has been reported to actively “nibble” OS particles from the OS tips before internalizing them [7, 8]. After internalization, the newly formed OS phagosomes are moved from the apical to the basal region inside the RPE, and simultaneously, OS phagosome maturation occurs as they interact with acidic endosomes and lysosomes [3, 4, 9, 10]. Eventually, the OS phagosomes are degraded in the RPE. Current knowledge on the process from uptake to degradation is mainly based on rods, and little is known about the regulation of cone OS phagocytosis.

OS tip phagocytosis is a daily occurring process with one or two peaks, traditionally defined by significant increases in the numbers of phagocytosed OS particles during a 24 h diurnal cycle depending on the animal species and their rod/cone ratio [11, 12]. In many rod‐dominant nocturnal animals, such as rat ( *Rattus norvegicus* ), Syrian hamster ( *Mesocricetus auratus* ), and northern leopard frog (*Rana pipens*), the burst of phagocytosis has been shown to occur once per day after light onset [13, 14, 15]. Interestingly, in more recent studies, either one or two peaks in OS phagosome numbers have been detected in rod‐dominant mice, depending on the mouse strain ([16, 17, 18, 19], Figure 3A). or diurnal cone‐dominant animals, such as Sudanian grass rat ( *Arvicanthis ansorgei* ) and zebrafish ( *Danio rerio* ), two peaks have been observed ([18, 20], Figure 3A). Recently, the field has recognized that these peaks in phagosome numbers are influenced not only by the rate of OS phagocytosis, but also by the rate of phagosome degradation in the RPE, with separate daily rhythmicities in the phagosome degradation and phagocytosis [21]. The peaks of OS phagosome numbers are also often associated with the change in light condition, especially in cone‐dominant animals. For example, in 14–16 *days post fertilization* (dpf) zebrafish, the first peak appears 4 h after light onset in the morning, whereas the second peak emerges 3 h after light offset in the evening [18, 22]. Additionally, the dependence of the process on the endogenous circadian clocks, the intrinsic timing mechanisms driving the day–night cycles of organisms' physiology [23, 24], has been investigated. In many mammals, such as mice and rats, the rhythmic nature of the process remains in constant darkness, demonstrating circadian regulation [25, 26]. However, in some vertebrates, such as frogs and goldfish ( *Carassius auratus* ), the peaks of OS phagosomes disappear when the ambient light is removed [27, 28], suggesting that the rhythm is driven primarily by the external light–dark cycle. Similar preliminary evidence is received from 16 dpf zebrafish; the peaks disappear in constant darkness [22]. However, it is worth noting that the Moran et al. data is based on three time points only, and thus do not take into account the possibility that removing the external light might shift the peak time of phagocytosis toward earlier or later hours of the day.

**FIGURE 1:**
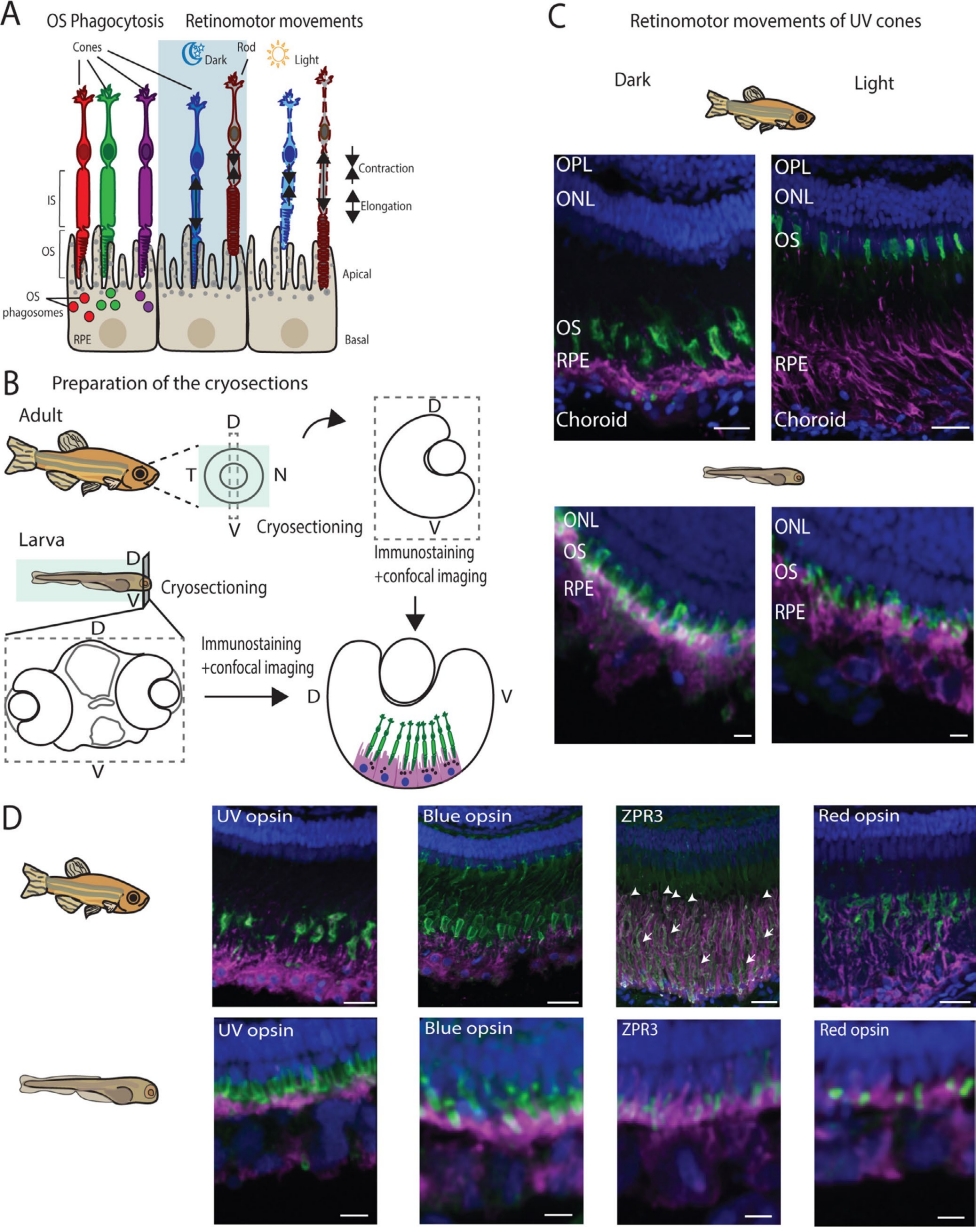
Adult and larval zebrafish retinal cryosections show structural differences at the photoreceptor–RPE interface. (A) Illustrative figure of the RPE–photoreceptor interface, outer segment (OS) phagocytosis and OS movement during the retinomotor movements in dark and light. Not to scale. (B) Illustrative figure of preparation and immunolabeling of adult and larval transverse retinal cryosections along the dorsoventral axis. Not to scale. D, dorsal; V, ventral; N, nasal; T, temporal. (C) Confocal images of adult (up) and larval (below) zebrafish retinal cryosections showing the interface between the RPE and UV cones in dark and light conditions. Adult cryosections show robust retinomotor movements seen as elongation and contraction of the UV cone OSs in the dark and light, respectively. Similar movements are not seen in larval cryosections (scale bars in the adult sections 20 μm; in the larval sections 5 μm). (D) Confocal images of adult (upper row) and larval (lower row) zebrafish showing immunolabeling of each cone subtype together with the RPE. Scale bars in the adult sections 20 μm and in the larval sections 5 μm. Green, outer segments of the photoreceptors; Magenta, RPE; Blue, Nuclei; Arrows, rods; Arrow heads, green cones; IS, photoreceptor inner segment; ONL, outer nuclear layer; OPL, outer plexiform layer; OS, outer segment; RPE, retinal pigment epithelium.

Most of the studies regarding the OS phagocytosis are conducted in animals with mature retinas [18, 20, 26, 29], leaving characteristics of the process in young animals with developing retinas elusive. In fact, 14–16 dpf old zebrafish were previously used to reveal the daily peaks of OS phagosomes [18, 22], but the rhythmic variations have neither been studied in younger nor older zebrafish, where the retina is at different developmental stages. When the zebrafish retina has reached its anatomical maturity at 20 dpf, retinal cross‐sections and flat mounts reveal multilayer organization of OSs of different photoreceptor types: four types of cones (ultraviolet (UV), blue, green, and red) and one type of rods [30, 31]. Instead, in larval zebrafish, the photoreceptors are approximately in the same layer up to 8 dpf [30, 31]. The OSs of different photoreceptor types can be distinguished from 4 dpf onwards [32], red/green double cones start to appear at 12 dpf, and all cone subtypes reach their full adult dimensions at 15 dpf [31]. Rods become mature even later, at 20 dpf [31, 33, 34]. As zebrafish develop, also the configuration of the photoreceptor OSs evolves continuously towards more organized conformation. Simultaneously, the physical interaction and the interactive processes between the photoreceptors and the RPE become more evident as the animal grows. An example of these interactive processes is retinomotor movements, seen as changes in photoreceptor OS positioning in relation to RPE tissue due to the light and circadian rhythms in teleost fishes ([35, 36], Figure 1A). These movements of rods and cones are converse: in the light, rods elongate deeper in the RPE while cones contract, and in dark conditions, rods contract while cones elongate ([36], Figure 1A). In contrast to adults, retinomotor movements of rods and cones are weak in the larval zebrafish retina under 28 dpf [35].

In the present study, we investigated variations in the diurnal rhythmicity of the OS phagosome numbers of the different cone subtypes using the young cone‐dominant zebrafish larva as an animal model. Our results demonstrate the presence of phagosomes from all cone subtypes throughout the day, with UV and blue cones showing two daily peaks. We also examined the effect of the ambient light on the process. Our results show that removal of the external light suppresses the rhythmic increase of the OS phagosome numbers, indicating that the daily peaks are primarily controlled by the ambient light in developing zebrafish.

## 2 Materials and Methods

### 2.1 Zebrafish Husbandry and Maintenance

Wild‐type zebrafish ( *Danio rerio* ) larvae at 7 dpf were used in the experiments as they have fully functional cone cell‐mediated perception. The embryos and larvae were grown in 10 cm petri dishes in E3 embryo solution (5 mM NaCl, 0.17 mM KCl, 0.33 mM CaCl_2_, 0.33 mM MgSO_4_, 0.0003 g/L Methylene Blue, pH 7.2) supplemented with 0.0045% 1‐Phenyl 2‐thiourea (PTU, Sigma‐Alrich, St. Louis, MO, USA) and maintained at 28.5°C in an incubator. Larvae were raised under a normal 14 h/10 h light–dark cycle (LD), unless otherwise stated, and were fed once a day with GEMMA Micro 75 (Skretting, Stavanger, Norway). Male and female animals were randomly chosen for each experimental group. For sample collection, zebrafish larvae were euthanized with an overdose (0.08%) of an anesthetic Tricaine (3‐aminobenzoic acid ethyl ester, pH 7.0) (Sigma‐Aldrich) prepared in E3 embryonal solution.

One‐ to 1.5‐year‐old adult Albino (slc45a2^ti225^) zebrafish (European Zebrafish Resource Center, Eggenstein‐Leopoldshafen, Germany, Research Resource Identification number, RRID: ZFIN_ZDB‐GENO‐120316‐69) were used for making histological cryosections. The fish were housed in a flow‐through water circulation system (at 25°C) under LD. Male and female animals were randomly chosen for cryoblock preparation. Fish were euthanized with an overdose (0.08%) of Tricaine.

### 2.2 Ethics Statement

The wild‐type zebrafish ( *Danio rerio* ) with AB/Tübingen background line, as well as the homozygote Albino (*slc45a2* ^*ti225*^) with Tübingen background line were obtained from Tampere Zebrafish Core Facility (Tampere University, Finland). Zebrafish were maintained according to the standard protocols and the ethical guidelines set by the Ethical Board in Finland. The husbandry and the experiments were done in accordance with the Finnish Act on the Protection of Animals Used for Scientific or Educational Purposes (497/2013) and the EU Directive 2010/63/EU. The ethical permissions for the experiments were granted by the Animal Experiment Board in Finland (the licenses ESAVI/37052/2020 and ESAVI/46385/2023).

### 2.3 Light Cycle Experiments and Sample Collection

To study the variation in the OS phagosome number, 7 dpf old larvae that were raised in 28.5°C in an incubator with a normal light cycle (14 h light/12 h dark; LD) were used. During the time point experiments, at 6 dpf, the larvae were transferred to room temperature (RT) at least 24 h prior to sample collection to match the environmental temperature with the temperature at which later dark‐adaptation experiments were performed. To maintain the LD light cycle at RT, an extra spotlight was utilized in the laboratory, and for the dark period, the larvae were placed in an opaque box. Larval samples were collected and euthanized at seven different time points over a 24 h period following light onset. Sample collection time points are expressed in Zeitgeber time (ZT) and were as follows: ZT1, ZT3, ZT5, ZT10, ZT16, ZT18, ZT23 (Figure 3B), where ZT0 is the light onset and ZT14 is the light offset. Larvae that were collected during the dark period at ZT16, ZT18, and ZT23 were euthanized in a dark room to avoid the influence of external light. Furthermore, to study the effect of external light on the peaks of OS phagosome numbers within the RPE, a separate group of 6 dpf larvae were dark adapted at least 24 h prior to sample collection. These larvae were kept in a dark–dark cycle (DD) by placing them into an opaque box in a dark room at RT. Larvae were collected at the same seven time points (ZT1, ZT3, ZT5, ZT10, ZT16, ZT18, ZT23) as previously described and euthanized in a dark room during sample collection.

### 2.4 Cryoblock Preparation and Cryosectioning

For cryoblock preparation of adult zebrafish samples, the fish were euthanized with Tricaine prior to enucleation. Collected eyes were then immersion‐fixed in 4% paraformaldehyde (Sigma‐Aldrich) in phosphate‐buffered saline (PBS) for 2 h at RT, followed by three PBS washes of 5 min each. For cryoblock preparation of larval zebrafish, larvae were collected at the time points mentioned above and euthanized with Tricaine prior to immersion‐fixation with 4% paraformaldehyde in PBS for 2 h at RT or overnight at +4°C, followed by three 5 min PBS washes. After fixation, larvae and adult zebrafish eyes were sucrose‐protected by incubating the samples in a sucrose gradient with rising concentrations (5%, 10%, 20%, 30%) for 1 h in each solution at RT, except for the 30% sucrose solution in which the incubation lasted for 24 h at +4°C. After sucrose‐protection, the larvae and adult eyes were embedded in tissue‐freezing medium (Tissue‐Tek O.C.T. compound; Sakura Finetek, Torrance, CA, USA) and frozen with liquid nitrogen. Larval cryoblocks were then sectioned with a cryotome (MEV+ cryostat; SLEE Medical GmbH, Nieder‐Olm, Germany) along the dorsoventral axis of the larval head to obtain 10‐μm‐thick cross‐sections including both larval eyes. Similarly, enucleated adult eye cryoblocks were sectioned to obtain 10‐μm‐thick eye cross‐sections. Cryosections were attached to adhesive microscope slides (Epredia Superfrost Plus Adhesion Microscope Slides; Fischer Scientific, Waltham, MA, USA) and incubated at 60°C–62°C for 2 h to ensure proper drying and attachment to the slides.

### 2.5 Immunohistochemistry

Cryosections were immunolabeled for opsin type‐ and RPE tissue‐specific proteins to observe OS phagosomes, originating from certain cone type, inside the RPE tissue. The binding sites of the used antibodies are listed in the supplementary table (Appendix S1, Table S1). Prior to immunolabeling, the sections were washed with 1× Tris‐buffered saline (TBS)‐Tween (0.05% of Tween) and permeabilized by incubating them in 0.05% Triton‐x‐100 in TBS‐Tween for 15 min and subsequently blocked with 3% bovine serum albumin (BSA) (Sigma‐Aldrich) in TBS for 1 h at RT. All primary and secondary antibody dilutions were prepared in 3% BSA‐TBS. Primary antibodies with the following concentrations were used in this study: antizebrafish Blue opsin (1:300), antizebrafish UV opsin (1:300), antirhodopsin [1D4] (1:100), zpr‐3 (1:200), anti‐RPE65 [N1C3] (1:300), zpr‐2 (1:200), antiopsin (1:200) (Table 1). Cryosections were incubated with primary antibodies for 24 h at +4°C followed by two TBS‐Tween washes and 24 h incubation with secondary antibodies (1:200) and phalloidin (1:100) (Table 2) in the dark. After secondary antibody incubation, the samples were washed twice with TBS‐Tween and once with MilliQ water followed by DAPI in MilliQ water (1:1200) labeling for 8 min at RT in the dark. Prior to mounting the samples with ProLong Diamond antifade mounting medium (Thermo Fischer, Waltham, MA, USA), the samples were washed once with MilliQ water.

**TABLE 1:** Primary antibodies with their target cell types, manufacturers, Research Resource Identification (RRID) numbers, and animal species in which the antibodies are produced.

Primary antibodies					
Antibody	Labeled cells	Manufacturer	Catalog number	Animal	RRID numbers
UV opsin	UV cones	Kerafast (Boston, MA, USA)	EJH013	Rabbit	AB_3674775
Blue opsin	Blue cones	Kerafast (Boston, MA, USA)	EJH012	Rabbit	AB_3674776
Opsin	Rods	Sigma‐Aldrich (St. Louis, MO, USA)	O4886	Mouse	AB_260838
Rhodopsin [1D4]	Red cones in zebrafish [37]	Abcam (Cambridge, UK)	ab5417	Mouse	AB_304874
zpr‐3	Green cones+Rods [38]	ZIRC (Eugene, OR, USA)		Mouse	AB_10013805
RPE65	RPE	GeneTex	GTX103472 (Irvine, CA, USA)	Rabbit	AB_2037911
zpr‐2	RPE	ZIRC (Eugene, OR, USA)		Mouse	AB_10013804

**TABLE 2:** Secondary antibodies, phalloidin, and DAPI with their targets, manufacturers, and Research Resource Identification (RRID) numbers.

Secondary antibodies, phalloidin, and DAPI				
Antibody	Target	Manufacturer	Catalog number	RRID number
Goat anti‐Mouse IgG (H + L) Highly Cross‐Adsorbed Secondary Antibody, Alexa Fluor 488	Mouse primary antibodies	Thermo Fischer Scientific (Waltham, MA, USA)	A‐11029	AB_2534088
Goat anti‐Rabbit IgG (H + L) Cross‐Adsorbed Secondary Antibody, Alexa Fluor 488	Rabbit primary antibodies	Thermo Fischer Scientific (Waltham, MA, USA)	A‐11008	AB_143165
Goat anti‐Rabbit IgG (H + L) Cross‐Adsorbed Secondary Antibody, Alexa Fluor 568	Rabbit primary antibodies	Thermo Fischer Scientific (Waltham, MA, USA)	A‐11011	AB_143157
Goat anti‐Mouse IgG (H + L) Highly Cross‐Adsorbed Secondary Antibody, Alexa Fluor 568	Mouse primary antibodies	Thermo Fischer Scientific (Waltham, MA, USA)	A‐11031	AB_144696
Phalloidin 643	F‐Actin (cell cytoskeleton)	ATTO‐TEC (Siegen, Germany)	ad643‐81	
4′,6′‐diamidine‐2‐phenylindole dihydrochloride (DAPI)	DNA (nuclei)	Sigma‐Aldrich, St. Louis, MO, USA	10 236 276 001	

### 2.6 Confocal Microscopy and Image Processing

All samples were imaged with a Nikon A1R laser scanning confocal microscope mounted in an inverted Nikon Ti‐E body (Nikon, Tokyo, Japan). A Nikon Apo 40×/1.15 DIC (Water) objective was used to image larval whole‐eye cryosections, and a Nikon Apo 60×/1.40 DIC (Oil) objective was used to image specific areas of larval and adult eye cryosections. 1024 × 1024‐pixel large *Z*‐stacks were taken to image the whole eyes (larvae) or specific retinal areas (adults and larvae) using 405, 488, 561, and 640 nm channels for fluorescent DAPI, RPE‐specific protein, photoreceptor‐specific opsin proteins, and phalloidin, respectively.

The imaging data was saved in .czi format and images were processed with ImageJ‐Fiji‐64 bit software (RRID: SCR_003070). Only linear brightness and contrast adjustments were performed for the pixel intensities in the images. Gaussian filtering with radius 1 (*σ* = 1) was done to improve the image quality when required. Final figures and graphs were constructed with Adobe Illustrator.

### 2.7 Analysis Tool for Quantitative Analysis of OS Phagosomes

The detection of OS phagosomes was performed using a custom‐developed semi‐automatized analysis tool for ImageJ‐Fiji (ImageJ 1.54) (Appendix S1, uploaded to Dryad repository). The tool utilizes standard ImageJ‐Fiji libraries for general image processing and MorphoLibJ (MorphoLibJ_‐1.6.2) for all morphological operations.

The analysis tool takes confocal *z*‐stack images of fluorescently labeled cryosections containing RPE and photoreceptor channels as input. Before further analysis, the stacks are processed into maximum intensity projections and denoised using a Gaussian filter (*σ* = 1). The entire workflow was executed as an interactive script that allows the user to adjust parameters as needed. First, a user‐defined RPE area is binarized through auto‐thresholding using Li's method [39]. A morphological opening with a disc‐shaped structuring element of radius 6 pixels is then applied to remove holes and smooth the selection. The final selection is saved as an ImageJ region of interest (ROI) to be used as the area to search for OS phagosomes in subsequent steps. OS phagosome segmentation is based on the extraction of local bright objects using the *h*‐dome transform, first introduced by Vincent (1993) [40] and since used for spot‐detection tasks in fluorescent and electron microscopy (EM) datasets [41, 42], to detect all local maxima. The *h*‐dome transform method suppresses low‐contrast regions and enhances significant intensity peaks bigger than the defined *h*‐value. The Maxima Finder is then applied to detect the intensity peaks with an additional criterion called prominence that is the minimum height difference between a local maximum and its surrounding region for the maximum to be considered significant.

For analysis tool development and parameter testing, a test set of *Z*‐stacks with each cone subtype labeled individually along with the RPE tissue was used. Empirical testing was conducted to determine appropriate values for the parameters *h*‐value and prominence to find the balance between sensitivity (detecting enough peaks) and specificity (avoiding false positives or irrelevant peaks) (Appendix S1, Text S1). The test analysis for each image was performed with the analysis tool using all combinations of five *h*‐dome values (5, 15, 25, 50, 100) and five prominence thresholds (5, 10, 20, 40, 80). The local maxima detected by the analysis tool were then matched to the manually annotated local maxima (ground truth) and the detected peaks were defined as true positive (TP), false positive (FP), and false negative (FN) as follows: A detected peak was considered a TP if it was within three pixels of a ground truth peak, a detected peak that could not be mapped to any ground truth peak was classified as a False Positive (FP), and a ground truth peak that was not mapped to any detected peak was classified as a false negative (FN). By using the counts of TP, FP, and FN, the values for precision, sensitivity, F1‐score, and false‐positive rate (FPR) (Defined in Appendix S1, Text S1) were calculated for each parameter combination of *h*‐values and prominence thresholds to evaluate the algorithm's performance and its overall detection reliability in the current analysis (Appendix S1, Text S1).

Since the parameter combinations of all five *h*‐dome values and prominence thresholds between 5 and 20 produced relatively similar values for precision, sensitivity, F1‐score, and FNR (Appendix S1, Text S1), the performance and peak detection reliability with these parameters were highly equal. Therefore, in our analysis tool, the *h*‐value was set to 15, and the prominence threshold was set specifically (always under the value of 20) for each analyzed image during the OS phagosome analysis to reach the most reliable detection of OS phagosomes. All analysis tool results were carefully inspected, and when the tool failed to detect the appropriate number of OS phagosomes, the number of phagosomes was quantified manually.

### 2.8 Quantitative Analysis and Statistical Testing

**FIGURE 2:**
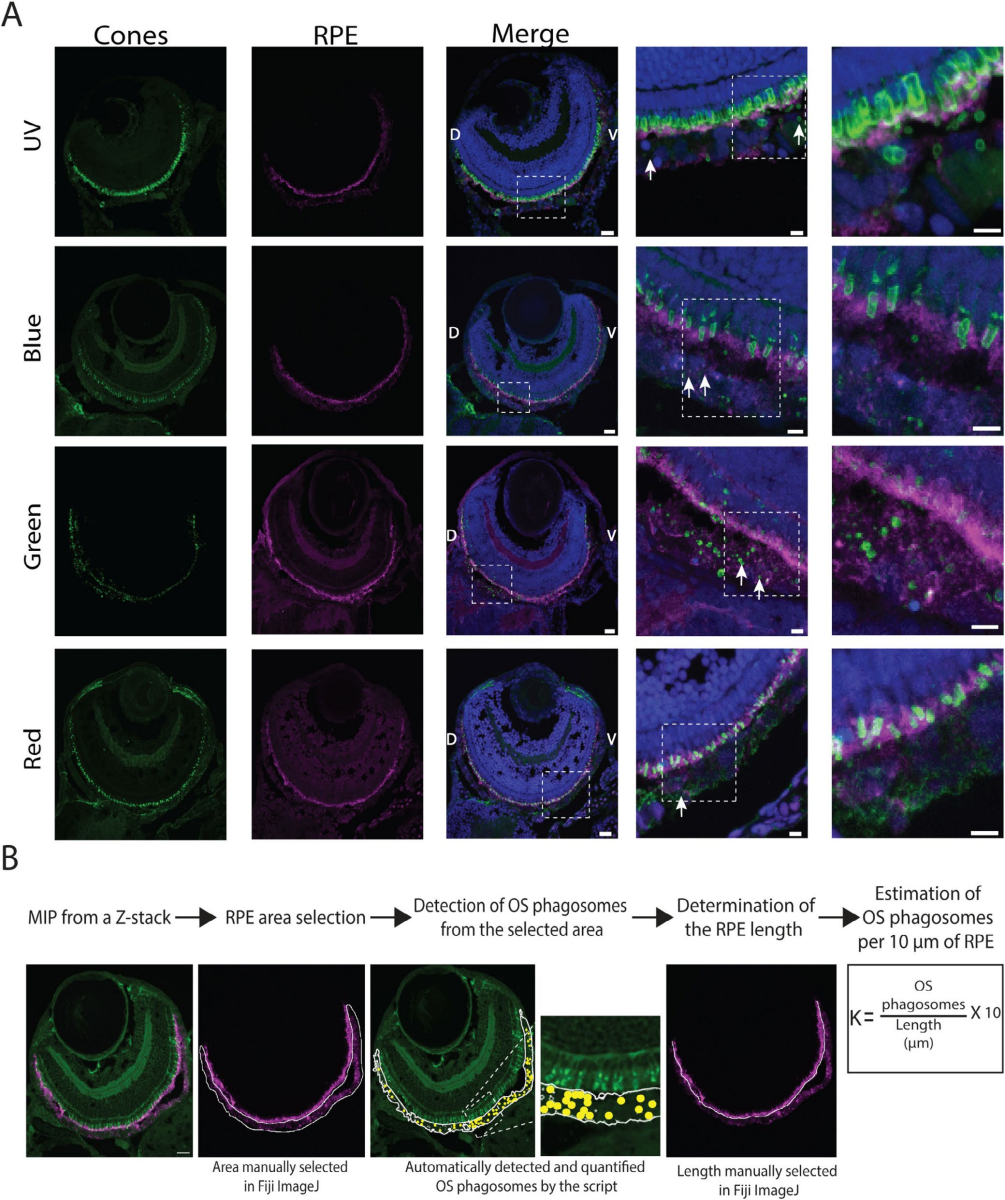
OS phagosomes from all cone subtypes can be detected and quantified from cryosections of the 7 dpf old zebrafish larvae. (A) Immunolabeling of cone subtype‐specific opsins and RPE tissue in the 7 dpf larval cryosections reveal OS phagosomes (white arrows) inside the RPE and verify that OSs of all cone subtypes (UV, blue, green, and red) are phagocytosed in the developing zebrafish. Scale bars in the whole‐eye cryosections 20 μm and 5 μm in the zoomed‐in views marked as white dashed boxes. (B) Operational steps of the semi‐automatized analysis tool for quantification of OS phagosomes from the selected RPE area in the larval cryosections as well as for defining the number of OS phagosomes per 10 μm of RPE tissue. Yellow circles represent the detected OS phagosomes. RPE, retinal pigment epithelium; D, dorsal; V, ventral; MIP, maximum intensity projection; OS, outer segment; K, Number of OS phagosomes per 10 μm of RPE.

**FIGURE 3:**
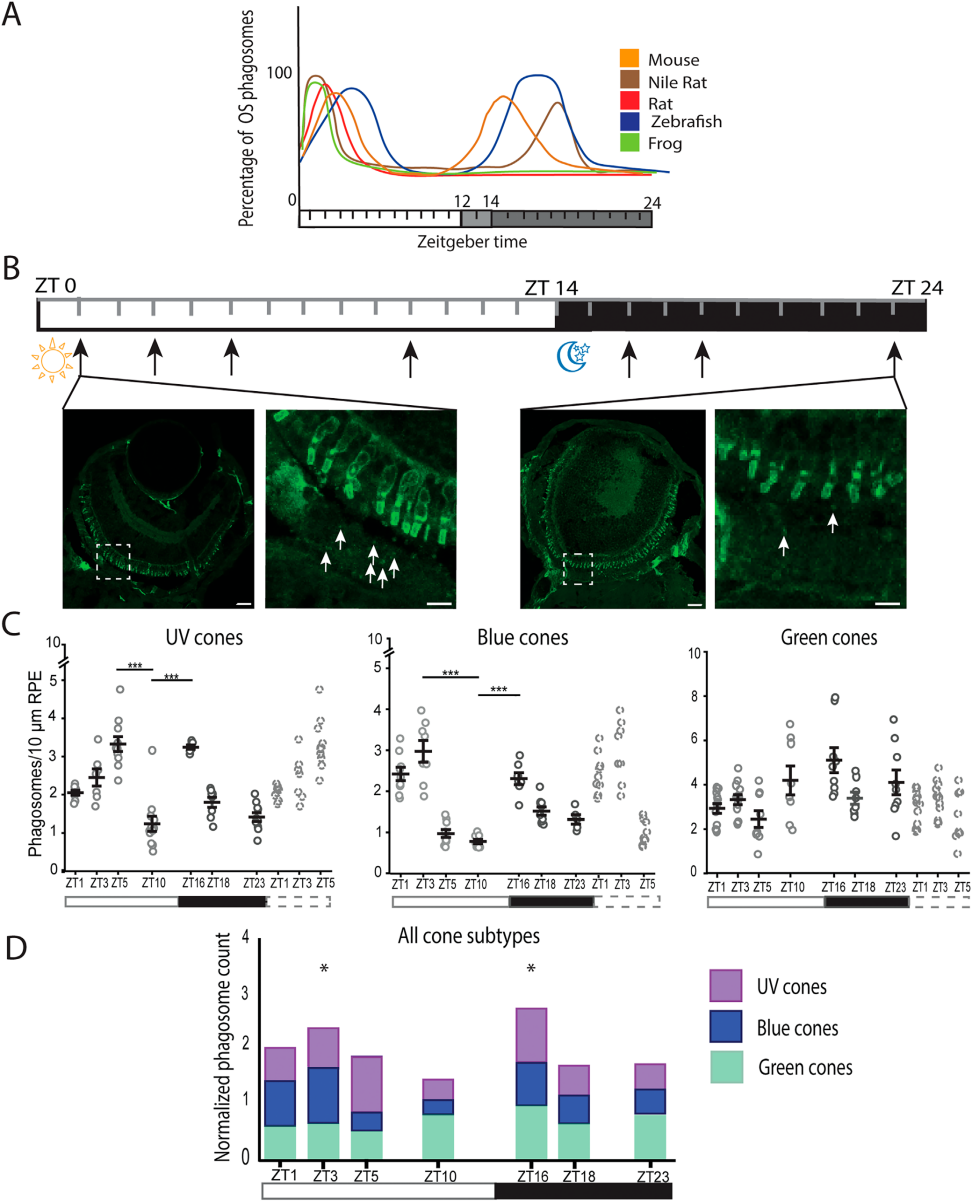
Number of phagosomes from the UV and blue cone OSs peaks twice during the 24 h cycle in the zebrafish larvae. (A) Illustrative figure of previous data generally demonstrates one or two peaks of OS phagosomes for various species. Figure is created after Moran et al. [11]. (B) 7 dpf zebrafish larvae were collected at seven time points (black arrows) and used for the cryosection preparation. After immunolabeling the sections for cone subtype‐specific opsins, the numbers of OS phagosomes were quantified. The differences in the numbers of phagosomes from blue cone OSs (white arrows) at two time points, ZT1 and ZT23, were evident in the zoomed‐in views (areas marked with dashed boxes). Scale bars in the whole‐eye sections are 20 μm and in zoomed‐in views 5 μm. (C) Scatter plots show quantitative results of phagosome numbers of UV, blue, and green cone OSs during the 24 h cycle. White and black bars show the light (ZT0‐ZT14) and dark (ZT14‐ZT24) phases of the day, respectively. Dashed bar shows the repeated three first sample collection time points. Numbers of phagosomes from UV and blue cone OSs showed rhythmic variation and peaked two times per 24 h, whereas the numbers of phagosomes from green cone OSs were more constant throughout the day. Data show the mean ± SEM. *p < 0.05, ***p < 0.001. (D) Stacked bar graph containing normalized data from UV, blue and green cones shows two peaks in OS numbers (marked with asterisks), and that UV cones and blue cones affect the rhythmic profile more than green cones. Additional graphs supporting this result are shown in Appendix S1, Figure S4. ZT: Zeitgeber time, OS, outer segment; LD, light–dark condition.

**FIGURE 4:**
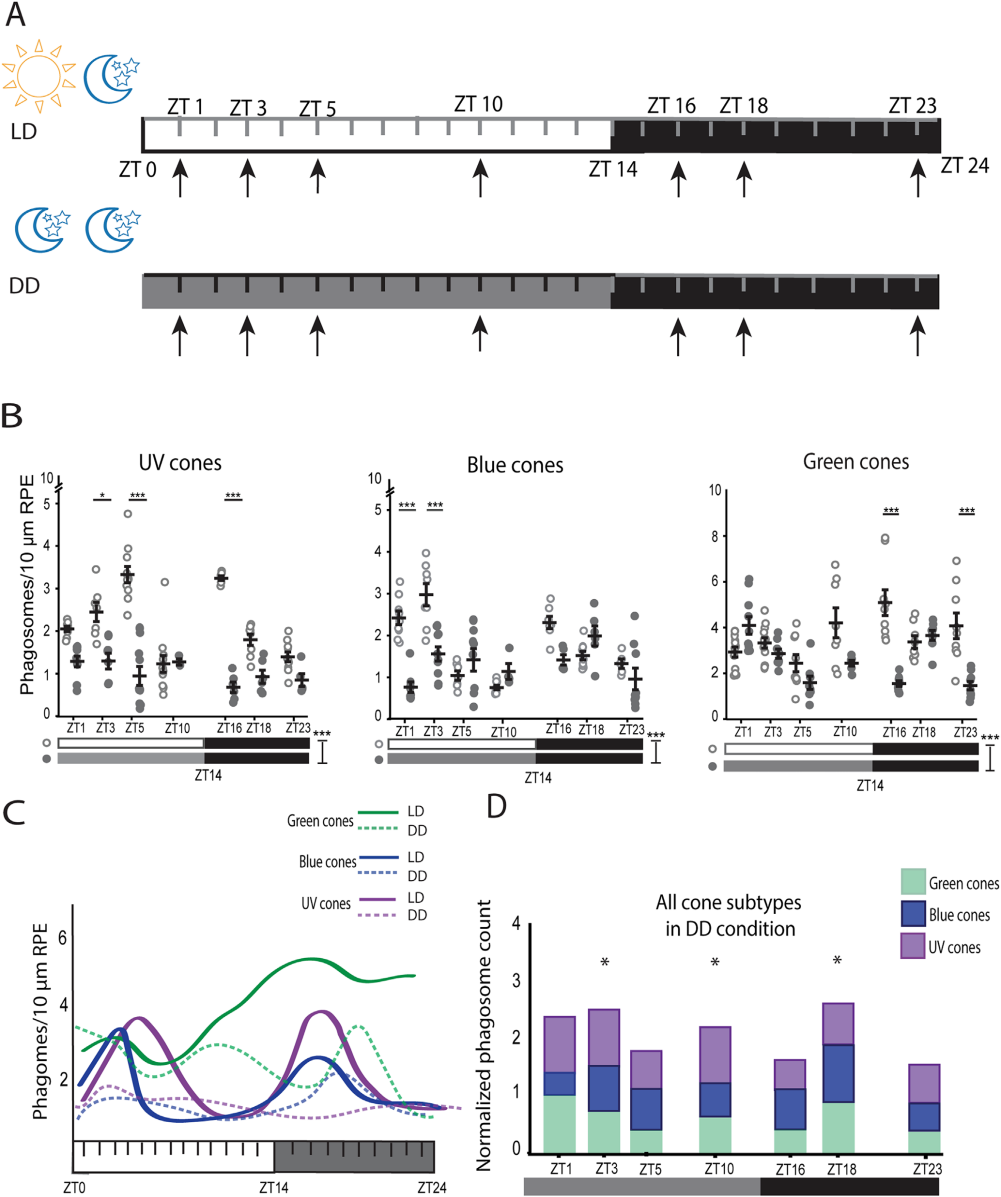
Peaks in the numbers of phagosomes from the UV and blue cone OSs dampen in constant darkness. (A) Zeitgeber timelines showing sample collection times (black arrows) in normal light cycle, LD (upper) and in constant darkness, DD (lower). (B) Scatter plots show phagosome numbers from UV, blue and green cone OSs during the 24 h cycle in LD (light circles) and in DD (dark circles) cycles. In addition, the peaks in the numbers of phagosomes from UV and blue cone OSs significantly dampened in DD compared to LD. Data show the mean ± SEM. *p < 0.05, ***p < 0.001. (C) Illustrative figure of the OS phagosome numbers from UV, blue and green cones in LD and DD over the 24 h cycle. In DD condition, the clear daytime peak of UV and blue cone OS phagosomes diminished and the number of OS phagosomes from all three cone subtypes decreased compared to LD condition. (D) Stacked bar graph containing the normalized data from UV, blue and green cones show slight rhythmicity in constant darkness with three time points at which the number of phagosomes increased (asterisks). Additional graphs supporting this result are shown in Appendix S1, Figure S4. ZT, Zeitgeber time; OS, outer segment; LD, light–dark condition; DD, dark–dark condition.

For OS phagosome number analysis, 4–12 larval whole‐eye cryosections (indicated as “n” in the figure legends) from a total of 3–9 larval individuals per time point were analyzed so that each section represented an individual larval eye. The OS phagosomes found in the RPE tissue were quantified from the whole‐eye cryosections using the custom‐developed analysis tool described above. The numbers of OS phagosomes found in the RPE tissue were normalized to the RPE length, which was measured from the RPE‐OS interface individually in each cryosection (Figure 2B). The data values in the graphs 3C and 4B show the number of OS phagosomes per 10 μm of RPE as well as the mean for the total number of samples (Figures 3C and 4B). Error bars represent standard errors of the mean (SEM) (Figures 3C and 4B). Normality of data distributions were tested using the Shapiro–Wilk test.

#### 2.8.1 LD Cycle Experiments

Data for each cone subtype was considered as normally distributed. One‐way ANOVA was performed to determine whether there was a statistically significant difference in the mean of OS phagosome numbers across the time points (ZT1, ZT3, ZT5, ZT10, ZT16, ZT18, ZT23) (Figure 3). Subsequent Bonferroni post hoc test was performed to determine which of the time points differed from each other. *p* value < 0.05 was considered statistically significant. Finally, the OS phagosome numbers at the peak time points were compared with the baseline OS phagosome number to evaluate whether the differences were statistically significant (Figure 3C). For this study, the baseline time point (ZT10) was chosen based on previous data by Lewis et al. [18] that showed the lowest number of phagosomes at ZT10.5.

#### 2.8.2 LD vs. DD Experiments

Data in LD and DD was considered as normally distributed for each cone subtype. A two‐way ANOVA was performed to examine the effect of light condition (LD versus DD) over the time points (ZT1, ZT3, ZT5, ZT10, ZT16, ZT18, ZT23) on the numbers of OS phagosomes found in the RPE (Figure 4B). A subsequent Bonferroni post hoc test was performed to determine which of the time points differed from each other in their OS phagosome numbers between LD and DD conditions.

Figures 3D and 4D show the stacked bar graphs of combined phagosome numbers for UV, blue, and green cone OSs in LD and DD, respectively. For these, the numbers of OS phagosomes from each cone subtype were normalized to the level of the highest number of phagosomes (phagosomes/10 μm of RPE) over the studied time points. Then, the normalized values for UV, blue, and green cones were stacked on top of each other. All statistical tests in this study were performed with Origin 2019b 64 bit and IBM SPSS Statistics 29.0.1.0. Final graphs were created with Origin and Adobe Illustrator.

## 3 Results

### 3.1 Adult and Larval Zebrafish Retinal Cryosections Show Differences in the Photoreceptor‐RPE Interactions

The organization of photoreceptors is well characterized in both adult and larval zebrafish retinae. In adults, OSs of different photoreceptor types are organized into different layers, whereas in larvae, a similar multilayer organization of photoreceptors is absent ([30, 31], Appendix S1, Figure S1). Additionally, in adults, the interaction between photoreceptors and the RPE changes significantly in different light conditions and during circadian‐related processes, such as OS tip phagocytosis and retinomotor movements containing elongation and contraction of rods and cones ([3, 36], Figure 1A). Instead, in larvae under 28 dpf, retinomotor movements of photoreceptors are minimal [35] and their phagocytosis has not been thoroughly studied.

To study and compare OS phagocytosis in adults and larvae, we prepared histological cross‐sections along the dorsoventral axis from both adult and larval zebrafish eye cryoblocks (Figure 1B). Next, we immunolabeled RPE and photoreceptor cells in the sections by RPE‐specific antibody and photoreceptor type‐specific opsin antibodies (Figure 1B). We used 7 dpf old larvae for our studies, as all cone subtypes (UV, blue, green, and red) are detectable and functional already at 5 dpf [31, 32, 43]. In accordance with previous studies [35, 36], our confocal images show robust and clearly observable retinomotor movements of photoreceptors in adults, but not in the 7 dpf old larval zebrafish retina (Figure 1C). Indeed, the adult UV cone OSs extended more into the RPE tissue in the dark than in the light (Figure 1C, upper row), but similar movements were not seen in the larvae (Figure 1C, lower row). With respect to OS phagocytosis studies, multilayered organization, and retinomotor movements make it challenging to distinguish the OS phagosomes from the intact photoreceptor outer segment layer in the adult fish. Moreover, estimating the exact localization of the OS phagosomes inside the RPE at different time points is demanding, as it is hard to determine whether they are in the long apical protrusion structures or inside the cell body. Since the retinomotor movements are lacking from larval zebrafish, for our approach with cryosections and immunohistochemical labeling, larval zebrafish is a more suitable model.

In search of specific antibodies to the cone subtypes in zebrafish, we found no publicly available specific antibodies for green cone opsin, in contrast to antibodies specific for the other opsins. Therefore, to detect green cones, we used zpr‐3, an antibody that is shown to label OSs of green cones and rods in adult zebrafish [38]. Our confocal images of adult and larval zebrafish cryosections demonstrate that antibodies for UV opsin, blue opsin, and red opsin label only one retinal layer as they specifically target UV, blue, and red cones, respectively (Figure 1D, Appendix S1, Figure S1). On the contrary, adult zebrafish cryosections clearly display zpr‐3 labeling in two retinal layers demonstrating rod and cone OSs (Figure 1D). Rod OSs (arrows) highly overlap with the RPE microvilli that extend up to 60 μm from the nuclear layer of the RPE, whereas the green cone OSs (arrowheads) locate closer to the inner retina. However, cryosections of 7 dpf larval zebrafish exhibit zpr‐3 labeling only in one retinal layer (Figure 1D, Appendix S1, Figure S1). Since at this age fully functional rods are not formed yet [31], we believe the antibody reveals principally only OSs of the green cones.

The challenges faced with the rod/green cone labeling by zpr‐3 antibody, as well as with the OS phagosome detection caused by retinomotor movements in adult zebrafish, can be overcome using young zebrafish larvae. This makes larval zebrafish a potential model for our studies on phagocytosis of individual cone subtype OSs. In addition, since the phagocytosis process is necessary for the photoreceptor viability to maintain normal visual function, we studied if the rhythmicity of the OS phagosome peaks has developed already in young zebrafish larvae, where the organization of the photoreceptors and the entire retina are still under modification.

### 3.2 All Cone Subtypes Are Phagocytosed in the 7 Dpf Old Zebrafish Larva

A previous study by Lewis et al. [18] showed evidence of cone phagocytosis in 7 dpf old zebrafish larvae, but phagocytosis of the different cone subtypes has remained unexplored. Here, to investigate whether all the different cone subtypes are phagocytosed in young zebrafish larvae, we used the above‐described approach with cryosections, immunohistochemical labelling, and imaging to detect OS phagosomes, originating from different cone subtypes, in the RPE. Our confocal microscopy data show that phagosomes from UV, blue, and green cone OSs can be detected in the RPE (Figure 2A). Moreover, the phagosomes from the red cone OSs were observed in the RPE, although the red opsin label in the images had more background signal than the other opsin labels, making it more challenging to distinguish the OS phagosomes from the background. Nevertheless, our data verify that all cone subtypes are phagocytosed already in the 7 dpf old larvae (Figure 2A).

To evaluate the numbers of cone OS phagosomes at different time points, they were quantified from the cryosections. To facilitate efficient quantification of the OS phagosomes, we developed a semi‐automatized analysis tool (Appendix S1, Text S1). Internalized OS particles are highly heterogeneous in size, shape, and fluorescence intensity, due to their state of degradation by the RPE's endo‐lysosomal machinery. This heterogeneity posed a challenge for automated detection. Moreover, the unspecific labeling of surrounding tissues lowered the contrast between particles and the background, making manual quantification highly demanding. We selected a semi‐automatized approach to avoid the need for tedious training of more intricate detection systems and to allow easy adaptation of the workflow to different cone subtype samples. For that, we developed an analysis tool that uses confocal *z*‐stacks of fluorescently labeled larval whole‐eye cryosections containing RPE and photoreceptor channels as input. For an OS particle to be considered as phagocytosed, its label had to overlap with the RPE label. This requirement effectively divided the rest of the workflow into two sections: RPE area selection and OS phagosome segmentation (Figure 2B, Appendix S1, Text S1). After segmenting the RPE area manually, the analysis tool detected and quantified OS phagosomes found in that area. Figure 2B also shows how the total length of the RPE tissue in the images was manually defined and how the number of OS phagosomes (K) per 10 μm of RPE tissue was calculated in later experiments.

To verify the accuracy and specificity of the analysis method to detect the OS phagosomes within the selected RPE area, the OS phagosome numbers were counted also manually from the images. The numbers of OS phagosomes for UV, blue, and green cones were comparable when quantified by either way (maximum difference in the phagosome numbers 10.5%) (Table 3). Thus, the analysis tool showed its reliability in quantification of OS phagosomes from immunofluorescent labeled cryosections. However, the tool did not work optimally for the red opsin label, since the numbers of OS phagosomes differed notably when quantified manually (minimum difference in the phagosome numbers 58%, maximum difference 129%) (Table 3). Therefore, we did not use the analysis tool further for the quantification of red cone phagosomes, but instead, later quantifications were performed manually.

**TABLE 3:** Comparison of the total OS phagosome numbers from different cone subtypes at two time points quantified manually and using the semiautomatized analysis tool from the larval zebrafish cryosections.

Cone subtype	Time point	Manual quantification	Semiautomatized quantification	Difference in OS numbers (%)
UV	ZT16	139	146	5.0
UV	ZT1	91	83	8.8
Blue	ZT16	92	98	6.5
Blue	ZT1	106	99	6.6
Green	ZT16	224	219	2.2
Green	ZT1	152	136	10.5
Red	ZT16	34	78	129
Red	ZT1	41	65	58.5

Abbreviations: OS, outer segment; ZT, Zeitgeber time.

### 3.3 The Phagosome Numbers From UV and Blue Cone OSs Show Rhythmic Variation in 7 Dpf Old Zebrafish Larvae

The number of OS phagosomes within the RPE peaks once or twice per day depending on the animal species (Reviewed by [11], Figure 3A). To study the time‐dependent variation of OS phagosomes from the different cone subtypes under normal LD conditions, zebrafish larvae were collected at seven discrete time points shown at ZT timeline with black arrows (Figure 3B). The samples were prepared and imaged as previously explained. Figure 3B also shows larval whole‐eye section immunolabeled with blue cone subtype‐specific antibody at two different time points ZT1 and ZT23. The difference in the OS phagosome numbers is clearly visible in the images between the time points with more OS phagosomes at ZT1 compared to ZT23 (Figure 3B).

Phagosomes from UV, blue, and green cone OSs were quantified from the defined RPE area in the images using the developed analysis tool. As the tool did not work reliably with the red cones, the phagosomes from red cone OSs were quantified manually. Since the detection of these phagosomes from the background labelling was challenging, we consider red cone data more unreliable than the data of the other cone subtypes. Therefore, we concentrated on UV, blue, and green cones in our further study, and red cone data are shown only as Supporting Information (Appendix S1, Figure S2, Figure S5). Our data show that the numbers of OS phagosomes varied significantly between the distinct cone subtypes as well as between the discrete time points (Figure 3C, Appendix S1, Figure S2). We used one‐way ANOVA analysis to show statistically significant differences in phagosome numbers from UV, blue, and green cones over the 24 h (Table 4) (*p* = 1.69 × 10^−14^; *p* = 9.10 × 10^−15^; *p* = 7.75 × 10^−4^, respectively). Subsequent Bonferroni post hoc test revealed two time points for UV (ZT5 and ZT16) and blue cones (ZT3 and ZT16) at which the OS phagosome numbers were significantly increased (Figure 3C). The number of phagosomes from UV cone OSs gradually increased first after light onset peaking at ZT5 (3.33 phagosomes/10 μm of RPE), and secondly after light offset at ZT16 (3.25 phagosomes/10 μm of RPE). At both peaks, the number of phagosomes from UV cone OSs was significantly higher compared with the baseline at ZT10 (baseline 1.24 phagosomes/10 μm of RPE, differences *p* = 1.22 × 10^−12^ for ZT5 and *p* = 1.74 × 10^−9^ for ZT16). Similarly, the second peak of phagosomes from blue cone OSs appeared after light offset at ZT16 (2.29 phagosomes/10 μm of RPE), but the light onset‐associated peak occurred earlier, at ZT3 (2.97 phagosomes/10 μm of RPE). At both peaks, the numbers of OS phagosomes were significantly higher compared with the baseline at ZT10 (baseline 0.77 phagosomes/10 μm of RPE, differences *p* = 1.44 × 10^−12^ for ZT3 and *p* = 1.94 × 10^−7^ for ZT16). Instead, the numbers of phagosomes from green cone OSs showed less variation between the time points, and clear peaks were not observed (Figure 3C). This indicates that green cones are phagocytosed at a more constant level throughout the 24 h cycle compared with the other subtypes. Moreover, the green cone data showed higher numbers of OS phagosomes at each time point compared to the other subtypes (Figure 3C, Table 4). Interestingly, manually calculated phagosomes from red cone OSs showed only one peak that emerged at a late dark timepoint of the day, at ZT23 (1.93 phagosomes/10 μm of RPE), with significantly more OS phagosomes compared with the baseline at ZT10 (baseline 0.69 phagosomes/10 μm of RPE, difference *p* = 1.80 × 10^−6^) (Appendix S1, Figure S2). In addition, the total number of phagosomes from red cone OSs throughout the 24 h cycle was lower than that of the other cone subtypes (Table 4, Appendix S1, Figure S2). Lastly, in addition to cone immunolabeling, a set of larval cryosections were immunolabeled with anti‐opsin antibody (O4886), previously shown to label rods in various vertebrate species such as mouse, rat, duck, turtle, and goldfish [44, 45] to visualize immature rod precursors and to evaluate the numbers of phagosomes originating from them. Surprisingly, our results show that the rhythmicity profile of the rod precursor OS phagosome peaks corresponds well to that of green cones (Appendix S1, Figure S3).

**TABLE 4:** Averages of phagosomes from UV, blue and green cone OSs per 10 μm of RPE at each time point.

Time	UV cones	Blue cones	Green cones	Average from the sum of UV, blue, and green cone OSs
ZT1	2.05	2.42	2.93	2.53
ZT3	2.45	2.97	3.33	3.02
ZT5	3.33	0.97	2.45	2.38
ZT10	1.24	0.77	4.18	1.88
ZT16	3.25	2.29	5.10	3.65
ZT18	1.80	1.50	3.38	2.22
ZT23	1.41	1.31	4.09	2.36
Average of total OS phagosome count over 24 h	2.15	1.76	3.59	

*Note: n* ≥ 5 sections at each time point, each section represents one eye.

Abbreviations: OS, outer segment; ZT, Zeitgeber time.

To evaluate the rhythmicity profile of OS phagosome peaks for each cone subtype in relation to the combined cone OS phagosome profile, automatically quantified phagosome numbers (i.e., from UV, blue, and green cone OSs) were normalized to the level of the highest number of OS phagosomes (phagosomes/10 μm of RPE) over the studied time points individually for each cone subtype. This normalization was relevant, since the opsin‐specific antibodies used for the different cone subtypes vary in their labeling specificity and efficiency, probably affecting the absolute numbers of the quantified phagosomes. The stacked bar graph containing normalized values shows that the sum of the phagosomes from UV, blue, and green cone OSs peaked twice during the 24 h cycle (Figure 3D), which is in line with the previous zebrafish studies [18, 22]. The first peak was associated with light onset and occurred at ZT3, whereas the second peak occurred after light offset at ZT16. The data also indicate a larger impact of UV and blue cones than green cones on the overall rhythmicity profile as the UV and blue cone bars, as well as their combination, form one light‐associated and one dark‐associated peak (Figure 3D, Appendix S1, Figure S4). The non‐normalized values of phagosome numbers from UV, blue, and green cone OSs were then summed together at each time point and after averaging, the numbers of phagosomes at the peak time points were 3.02 phagosomes/10 μm of RPE and 3.65 phagosomes/10 μm of RPE at ZT3 and ZT16, respectively (Table 4, Appendix S1, Figure S4). Table 4 and Appendix S1, Figure S4 show that at both peak time points, the phagosome numbers were higher compared to the baseline at ZT10 (1.88 phagosomes/10 μm of RPE). Collectively, our results indicate that the numbers of phagosomes originating from the larval UV and blue cone OSs rhythmically increase twice a day, but similar rhythmicity is not observed with the green cones.

### 3.4 Rhythmic Changes in the Phagosome Numbers From UV and Blue Cones Reduce in Constant Darkness

Previous studies have shown that the rhythmic nature of OS phagosomes within the RPE is retained in a constant dark environment, indicating that the process is regulated by intrinsic circadian clocks in many species, such as mouse, rat, and Sudanian grass rat [20, 25, 26]. However, in some species, such as in frogs and goldfish, peaks in OS phagosome numbers disappear when the ambient light is removed [27, 28]. Here, to study this regarding the OS phagosome peaks for the different cone subtypes in zebrafish, the larvae were kept in constant darkness (DD) for at least 24 h before and up until sample collection (Figure 4A). Samples were collected at the same time points and treated and analyzed as previously described for the LD treated larvae. The numbers of phagosomes from UV, blue, and green cone OSs were quantified with the developed analysis tool, whereas OS phagosomes from red cone OSs were calculated manually.

A two‐way ANOVA analysis showed a statistically significant reduction in the phagosome numbers from UV, blue, and green cone OSs in DD compared to LD over 24 h (*p* = 5.87 × 10^−20^, *p* = 5.58 × 10^−5^ and *p* = 7.59 × 10^−8^, respectively) (Figure 4B). Subsequent Bonferroni post hoc analysis showed that the numbers of phagosomes from UV cone OSs at both peaks seen in LD (Figure 3C) were significantly lower in DD (ZT5: *p* = 6.58 × 10^−17^, ZT16: *p* = 1.55 × 10^−12^) (Figure 4B). Similarly, the first peak of phagosomes from blue cone OSs at ZT3 in LD was significantly dampened (*p* = 8.70 × 10^−6^) under DD light conditions. Additionally, the second peak of phagosomes from blue cone OSs was slightly dampened and shifted from ZT16 to ZT18. However, the difference in OS phagosome numbers between LD and DD at this new peak time point (ZT18) was not statistically significant (Figure 4B). The number of phagosomes from green cone OSs was also significantly decreased at the two time points, ZT16 and ZT23, in DD (ZT16: *p* = 3.65 × 10^−8^, ZT23: *p* = 6.86 × 10^−5^) (Figure 4B). Figure 4C summarizes how the rhythmic profiles of UV, blue, and green cone OS phagosomes found in the RPE alter in response to change in light conditions from LD (solid lines) to DD (dashed lines). The peaks in the numbers of phagosomes from UV and blue cone OSs clearly diminish in DD light conditions, but the profile of green cone phagocytosis remains more constant, as clear peaks were not seen even in LD (Figure 4C).

The two‐way ANOVA analysis showed that the change in light condition did not affect the total number of manually quantified phagosomes from red cone OSs throughout the 24 h (Appendix S1, Figure S5). However, the Bonferroni post hoc test revealed a significant reduction in red cone OS phagosomes at ZT23 when LD and DD conditions were compared (*p* = 1.42 × 10^−6^) (Appendix S1, Figure S5). Interestingly, at ZT3, the number of phagosomes from red cone OSs was significantly higher in DD than in LD (*p* = 5.60 × 10^−4^). A similar increase in OS phagosome numbers in DD at any time point was not seen with the other cone subtypes (Figure 4B).

To evaluate the contribution of UV, blue and green cone subtypes on the combined rhythmicity profile in DD condition, OS phagosome numbers for each of these cone subtypes were normalized and then summed together as described in Section 3.3. The stacked bar graph containing UV, blue, and green cone subtypes shows three time points during the 24 h cycle in DD condition at which the sum of the normalized values of these cone phagosomes peaked slightly (Figure 4D). Figure 4D shows that the supposed light onset‐associated peak of OS phagosomes emerged already at ZT1 and continued until ZT3. This was different from the LD condition, where the peak, containing the same cone subtypes, did not emerge until ZT3 (Figure 3D). In addition, in DD, the peak after the supposed light‐to‐dark transition appeared at ZT18 which was later than in LD at ZT16 (Figures 3D and 4D). Surprisingly, different from LD condition, a slight increase in OS phagosome numbers emerged also at ZT10 in DD, which seems to be most affected by the phagosomes from the UV cones (Figure 4D, Appendix S1, Figure S4). The non‐normalized values of UV, blue and green cone phagosomes were then summed and averaged at each time point (Table 5, Appendix S1, Figure S4). Altogether, the numbers of detected OS phagosomes over the 24 h were more constant compared to those in LD condition (Tables 4 and 5, Appendix S1, Figure S4).

**TABLE 5:** Averages of phagosomes from UV, blue, and green cone OSs per 10 μm of RPE at each time point in DD.

Time	UV cones	Blue cones	Green cones	Average from the sum of UV, blue and green cone OSs
ZT1	1.29	0.77	4.10	2.17
ZT3	1.30	1.56	2.86	1.93
ZT5	0.95	1.42	1.59	1.29
ZT10	1.29	1.15	2.42	1.78
ZT16	0.68	1.40	1.56	1.24
ZT18	0.93	1.97	3.67	2.34
ZT23	0.87	0.94	1.47	1.13
Average of total OS phagosome count over 24 h	1.04	1.30	2.56	

*Note: n* ≥ 5 sections at each time point, each section represents one eye.

Abbreviations: OS, outer segment; ZT, Zeitgeber time.

## 4 Discussion

Phagocytosis of photoreceptor outer segments has been studied intensively over several decades, focusing primarily on rods. However, the rhythmic nature and regulation of cone OS phagocytosis have remained more elusive, and to our knowledge, no previous studies regarding the differences between cone subtypes have been conducted. Since cone subtypes display morphological and functional differences, divergence may also exist in the rhythmicity of the process. In addition, as previous studies have been primarily conducted with adult animals [20, 26, 46], rhythmicity and regulation of OS phagocytosis are largely unresolved in developing animals. In the present work, we studied the cyclic occurrence of OS phagosome peaks of all individual cone subtypes (UV, blue, green, and red cones) in cone‐dominant 7 dpf old zebrafish larvae using immunohistochemistry, confocal microscopy, and semi‐automatized quantification of OS phagosomes in the RPE. We showed that all cone subtypes are phagocytosed in young, developing zebrafish and OS phagosomes from each subtype can be found within the RPE throughout the day. Interestingly, we observed two peaks in the numbers of OS phagosomes from UV and blue cone OSs over the 24 h, indicating already developed rhythmicity in the internalization and/or degradation phases of the OS particles. The daytime peaks of UV and blue cones disappeared, and the total number of phagosomes from all cone subtypes reduced in constant darkness conditions, indicating that external light has a significant influence on cone phagocytosis in young larval zebrafish.

Our data reveal that the phagocytosis of all cone subtypes is ongoing already in 7 dpf young zebrafish larvae (Figure 2A). As a comparison, in mice and rats, the rod OS phagocytosis process has been shown to begin around the time of eye opening: at Postnatal Day P14–P15 in mice and P15–P16 in rats [47]. In these rod‐dominant mammals, rhythmicity of the process develops and aligns with external light cycles as well as intrinsic circadian cycles during the subsequent days as the retina and circadian systems continue to develop [47]. Our data from 7 dpf zebrafish demonstrate two light/dark‐transition‐associated peaks in the combined phagosome numbers from UV, blue, and green cone OSs during the 24 h circadian cycle (Figure 3D, Table 4, Appendix S1, Figure S4). This result agrees with previous data combining either rods and cones or all cone subtypes without rods and obtained from various species, including certain mouse strains, such as (C57BL/6J), Sudanian grass rat, chicken (*Gallus domesticus*), domestic cat, rhesus monkey ( *Macaca mulatta* ), goldfish, and zebrafish [12, 17, 18, 20, 22, 48, 49, 50, 51]. However, when the different cone subtypes are examined separately, our quantitative data suggest that the daily variation in the numbers of OS phagosomes originating from the distinct cone subtypes is not identical. Based on our data, it seems that single cones (UV and blue) have stronger rhythmicity in the OS numbers than the future forming double cones (green and red) have (Figure 3C, Appendix S1, Figure S2, Figure S4). In Figure 3C, the two peaks in OS phagosomes from UV and blue cones indicate either more active internalization of the OS particles or a slower rate of OS phagosome degradation [21]. As shown by [21], the rates of internalization and degradation may contribute differently to the light onset‐associated peak and the peak seen after light offset: In mice, the morning peak is largely affected by a slower rate of OS phagosome degradation, whereas the evening peak results mostly from increased OS particle internalization. Although the kinetics of OS particle uptake and degradation in zebrafish may share similarities with mice, our data does not address how the changes in the rates of internalization and/or degradation affect the numbers of OS phagosomes at the studied time points.

Despite the ongoing discussion regarding the contribution of OS particle internalization and/or degradation to the peak formation, our result of the rhythmic variation of UV and blue cone phagosomes is supported by a previous study by Krigel et al. [52] that utilized the neural retina leucine zipper gene knockout mouse strain (*Nrl* ^*−/−*^) to study the rhythmicity of the phagocytosed OS particles from blue cones [52]. These mice completely lack rods in the retina, but the remaining photoreceptors seem to be identical to blue cones. Krigel et al. showed that in these mice, the rhythmic profile of the blue cone OS phagosomes corresponds to the rhythm of rods in wild‐type mice with a significant peak in OS phagosome numbers at 1 h after light onset and a slight increase at 1 h after light offset. While our results revealed a rhythmic nature for the occurrence of the UV and blue cone OS phagosome peaks, the green and red cones did not show similar rhythmicity profiles. We speculate this to be due to differences in the developmental phases of the cone subtypes in the larval zebrafish retina. It is commonly recognized that UV and blue cones develop and mature earlier than green and red cones [32, 53, 54, 55]. Additionally, in young zebrafish larvae, all cones appear as single cones, since the red/green double cones are visible only after 12 dpf [30, 31, 56]. Therefore, we assume the green and red cones to be less mature than the UV and blue cones at 7 dpf. More developed rhythm of UV and blue cones could also be related to the level of their activity in zebrafish at this age. As the main objective of the larvae is to survive and grow, prey capture is at the center of their behavior. In the larval zebrafish, the UV cones are known to be used primarily in prey detection [56, 57], highlighting their importance for the larvae. This could partially explain the higher demand for their rhythmic and synchronized renewal already in the young individuals.

In addition to the different rhythmic profiles of the OS phagosome peaks of the distinct cone subtypes, our data showed variation in the total numbers of cone type‐specific phagosomes over a 24 h cycle (Figure 3C, Table 4, Appendix S1, Figure S2, Figure S4). However, as the numbers can be affected by the used antibodies, it is not straightforward to compare the absolute numbers of OS phagosomes between the different cone subtypes. For instance, our results showing the higher number of green cone phagosomes compared with the other cone subtypes can be influenced by the used antibody. To our knowledge, an antibody specific to only green cone opsin in zebrafish is not publicly available. Therefore, we used zpr‐3, an antibody that has been shown to label the OSs of green cones and rods in zebrafish [38]. As fully functional rods do not appear before 20 dpf in zebrafish [31], it is likely that the demand for the renewal of rod precursor OS membranes is not remarkable in 7 dpf old larvae. However, we cannot neglect the possibility that rod precursors are renewed and phagocytosed to some extent, and this way they could affect our results showing higher numbers of quantified phagosomes labeled by zpr‐3. Interestingly, our immunolabeling showed that opsin (O4886) and zpr‐3 antibodies both in the adult and larval cryosections were similar, indicating that both antibodies label not only rods (in adults) or rod precursors (in 7 dpf old larvae) but also green cones in zebrafish (Appendix S1, Figure S3). Therefore, we were not able to specifically label rod precursors and evaluate how much they contribute to the observed phagosome numbers in the larval cryosections. We encountered antibody specificity issues also in the labeling of red cones, which could explain the low numbers of the detected phagosomes from red cone OSs in our cryosections. We used rhodopsin [1D4] antibody (ab5417, Abcam) that has been shown to label rods in the mouse retinal samples [58], but interestingly, [37] showed that in zebrafish, this antibody labels OSs of red cones [37]. In our experiments, this antibody showed noisy and unspecific labeling in the cryosections. Thus, the phagosomes from red cones were hard to distinguish from the background. We assume this to be one reason also for the failed quantification with the created semi‐automatized analysis tool, as it cannot detect and segment the OS particles of red cones properly from the background. Therefore, the analysis tool should be trained further for a more reliable quantification of OS phagosomes of this cone type. Furthermore, both rhodopsin (1D4) and zpr‐3 antibodies recognize and bind to an epitope at the C‐terminus of the rhodopsin protein ([38, 59], Appendix S1). The C‐terminus of the rhodopsin is digested rapidly within the RPE after OS particle internalization, as the formed OS phagosome is fused to acidic enzymes containing endosomes [60]. This makes the rhodopsin [1D4] and zpr‐3 antibodies useful only for detecting newly internalized OS particles. Since our UV and blue cone subtype‐specific opsin antibodies bind to the more stable N‐termini of their target proteins, the phagosomes both at early and later stages of digestion can be detected with these antibodies. Overall, since the used opsin‐specific antibodies differ in their binding sites on the opsin proteins as well as in their specificity, it is more meaningful to compare the rhythmicity profiles than the absolute phagosome numbers between the different cone subtypes.

As our results showed rhythmicity in the phagosome numbers of UV and blue cone OSs, we further studied the involvement of external light in the process. For that, we kept zebrafish larvae in constant darkness for a minimum of 24 h before sample collection, preparation, and OS phagosome analyses. These constant darkness experiments showed that the impact of the lack of external light on the cone OS phagosome numbers was evident in the larval zebrafish with a notable decrease in the total number of OS phagosomes over a 24 h cycle compared with the LD condition (Figure 4B, Tables 4 and 5). Moreover, in DD, the peaks in OS phagosome numbers from UV, blue, and red cones diminished, and the daytime peaks of UV and blue cones were completely abolished (Figure 4B,C, Appendix S1, Figure S5). This result differs from the previous study by Krigel et al. where they showed that the rhythmicity of the blue cone phagosome peaks remained in DD condition in the *Nrl* ^*−/−*^ mouse model and concluded that the phagocytosis of the blue cone OSs is under circadian control [52]. On the contrary, our data indicate that in young zebrafish larvae, the occurrence of the cone OS phagosome peaks is substantially driven by the alternating light–dark cycles and regulation by the intrinsic circadian system is less clear. This finding is also supported by the study of Moran et al. [22] from 16 dpf larvae. Overall, circadian rhythms related to zebrafish cellular functions and behaviors show differences in the maturation rates (Reviewed by [61]). It is thus plausible that the circadian regulation of the OS phagocytosis seen in mice, rats, and cultured RPE cells [29, 46, 62, 63] is still developing in the 7 dpf old zebrafish larvae and therefore is not observable at that age. Nevertheless, it is worth noting that in goldfish and frogs, the OS phagocytosis peaks are primarily caused by the light–dark cycles even in the adult stages [27, 28]. Zebrafish belong to the teleost class of fishes as does goldfish. Therefore, the process could be regulated by the light cycles also in the adult zebrafish.

In conclusion, this study showed that under a normal light cycle, all cone subtypes are phagocytosed in the young zebrafish larvae. Surprisingly, the occurrence of the OS phagosome peaks seems to be different for the distinct cone subtypes at 7 dpf. For the UV and blue cones, the rhythmicity is evident with two daily peaks of OS phagosomes, whereas for the green cones, clear rhythmicity was not observed. Definitive conclusions for the red cones could not be made from the quantitative data due to technical challenges with the antibody specificity. Additionally, our results revealed the influence of changes in light–dark conditions for the rhythmic peaks of OS phagosomes in the 7 dpf old larvae, suggesting weaker regulation by the intrinsic circadian clocks either related to developmental immaturity or species‐specific characteristics. Further research is needed to determine the mechanism by which the phagocytosis of the distinct cone subtype OSs is differentially regulated and to what extent the circadian system is involved.

## Author Contributions

Design and conceptualization of the study was done by Jenni Partinen, Noora Nevala, Soile Nymark and Teemu Ihalainen. Experimental work was performed by Jenni Partinen. Analysis and interpretation of the data was done by Jenni Partinen, Noora Nevala, Teemu Ihalainen, and Soile Nymark. Analysis tool was created and tested by Sanni Erämies. The first draft of the manuscript was prepared by Jenni Partinen, and all authors participated in the manuscript editing and finalizing. Funding for the project was obtained by Jenni Partinen, Noora Nevala, Teemu Ihalainen, and Soile Nymark.

## Conflicts of Interest

The authors declare no conflicts of interest.

## Supporting information


Appendix S1.

## Data Availability

The data that support the findings of this study will be openly available in the Dryad repository at https://doi.org/10.5061/dryad.76hdr7t64.
