# Supplementary material for: Phagocytosed Photoreceptor Outer Segment Particles Within the Retinal Pigment Epithelium Show Diurnal Rhythmicity and Variation Between Cone Subtypes in Larval Zebrafish
Source: FASEB J. 2025 Jul 24;39(14):e70853. doi: 10.1096/fj.202500211R (PMC12288107; doi:10.1096/fj.202500211R)
Supplement: Supplementary file 1 — Appendix S1. [file FSB2-39-e70853-s001.zip › fsb270853-sup0007-Figure S5.pdf]

Supplemental material

Figure S5

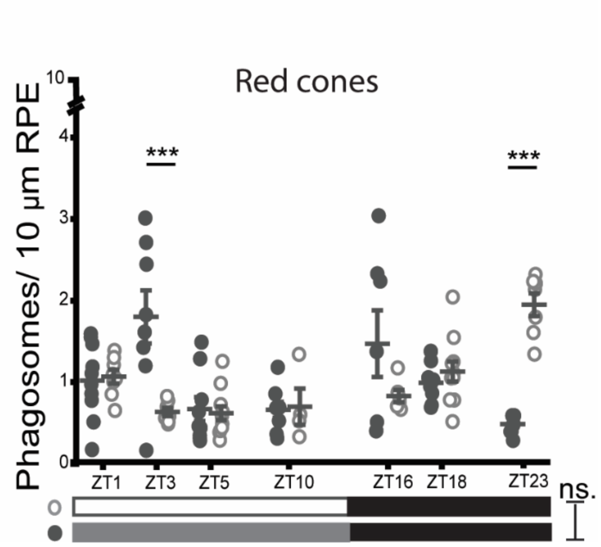

Time	Red cones in DD
ZT1	1.02
ZT3	1.80
ZT5	0.67
ZT10	0.65
ZT16	1.46
ZT18	0.98
ZT23	0.47
Average of total OS phagosome count over 24 hours	0.99

Fig S5. *The numbers of phagosomes from red cone OSs in the RPE* over the 24 hours in constant darkness.
The *scatter plot shows the numbers of phagosomes from red cone OSs per 10 µm of RPE at each studied time point in normal light cycle (LD, white) and in constant darkness (DD, Dark grey). Data is represented together with the mean line ± SEM. At ZT23, the peak seen in LD was diminished in DD (***p<.001), while a clear peak emerged at ZT3 (***p<.001). Two-way ANOVA analysis was used to show that there was non-significant (ns.) difference in the numbers of phagosomes from red cone OSs over the 24 hours between LD and DD conditions. The table shows averages of the phagosomes from red cone OSs per 10 µm of RPE at each time point under constant darkness. Phagosomes were quantified from the length of the entire RPE tissue in each whole eye section. N≥5 sections at each time point, each section represents one eye. RPE: Retinal pigment epithelium, ZT: Zeitgeber time, OS: outer segment.*
