# Supplementary material for: Phagocytosed Photoreceptor Outer Segment Particles Within the Retinal Pigment Epithelium Show Diurnal Rhythmicity and Variation Between Cone Subtypes in Larval Zebrafish
Source: FASEB J. 2025 Jul 24;39(14):e70853. doi: 10.1096/fj.202500211R (PMC12288107; doi:10.1096/fj.202500211R)
Supplement: Supplementary file 1 — Appendix S1. [file FSB2-39-e70853-s001.zip › fsb270853-sup0001-Table S1.pdf]

Supplemental material

Table S1

Table S1: Binding sites of the used opsin antibodies at their target opsin protein

Antibody	Labelled cells	Binding site at opsin protein, amino acids	Binding site at opsin protein, terminus
UV opsin	UV cones	1-27	N
Blue opsin	Blue cones	1-41	N
Rod opsin	Rods in various vertebrate phyla: mammal, avian, amphibian, fish (Barnstable 1980, Silver et al., 1988)	4-10	N
Rhodopsin [1D4]	Red cones in zebrafish (Yin et al., 2012)	339-348	C
zpr-3	Green cones+Rods (Hu et al., 2024)	320-354	C
