# Supplementary material for: Phagocytosed Photoreceptor Outer Segment Particles Within the Retinal Pigment Epithelium Show Diurnal Rhythmicity and Variation Between Cone Subtypes in Larval Zebrafish
Source: FASEB J. 2025 Jul 24;39(14):e70853. doi: 10.1096/fj.202500211R (PMC12288107; doi:10.1096/fj.202500211R)
Supplement: Supplementary file 1 — Appendix S1. [file FSB2-39-e70853-s001.zip › fsb270853-sup0002-Text S1.pdf]

Supplemental material

Text S1

A) The full script of the semi-automatized analysis tool for quantitative analysis of outer segment (OS) phagosomes in the RPE tissue.

```
/// Macro by Sanni Erämies (sanni.eramies@tuni.fi)
/// Version: 08.01.2025
/// Requires: MorphoLibJ, ResultsToExcel (test mode only)
/// Description:
/// This macro assists in particle analysis from RPE images using h-dome transformation
/// for peak detection. The process includes user interaction for parameter selection,
/// channel configuration, RPE area selection, and analysis based on provided parameters.
/// The results are saved as ROI files and optionally as Excel sheets for further analysis.
///
/// Key Features:
/// - User-driven RPE area selection.
/// - Automatic particle detection using h-dome values and prominence thresholds.
/// - Outputs ROIs of detected particles and RPE area.
///
/// Sections:
/// 1. Function Definitions
/// 2. Main Macro Execution
//////////////////////////////////////////

// Function Definitions

/// Displays a dialog for user input and initializes key paths and parameters.
///
/// Inputs:
/// - None (user provides input via a dialog).
///
/// Outputs:
/// - An array containing the following:
///   - `input` (`string`): The user-selected input directory.
///   - `output` (`string`): The user-selected output directory.
///   - `h_input` (`number`): The user-defined h-value for dome detection.
function startup(){
		Dialog.createNonBlocking("RPE particle analysis");
		Dialog.addDirectory("Input Directory", getDirectory("home"));
		Dialog.addDirectory("Output Directory", getDirectory("home"));
		Dialog.addNumber("Define h-value for dome detection", 15); // Default h-value for
dome detection

		Dialog.show();
		input = Dialog.getString(); // User-selected input directory
		output = Dialog.getString(); // User-selected output directory
		h_input = Dialog.getNumber(); // User-defined h-value

		return newArray(input, output, h_input);
}

/// Allows the user to define which channels to use for analysis.
```

```
///
/// Inputs:
/// - None (user selects channels via dialog).
///
/// Outputs:
/// - An array containing the following:
///   - `rpe_ch` (`string`): The user-selected RPE channel.
///   - `particle_ch` (`string`): The user-selected particle channel.
function channelCheck(){
		Dialog.createNonBlocking("Channels");
		Dialog.addChoice("RPE:", CHANNELS, 2);
		Dialog.addChoice("Particles:", CHANNELS, 3);
		Dialog.show();

		ch1 = Dialog.getChoice(); // RPE channel
		ch2 = Dialog.getChoice(); // Particle channel

		title = "MIP";
		run("Duplicate...", "duplicate channels="+ch1+"-"+ch2+" title="+title);
		close("\\Others");
		run("Split Channels");

		rpe_ch = "C1-"+title;
		particle_ch = "C2-"+title;

		return newArray(rpe_ch, particle_ch);
}

/// Enables the user to select the RPE area for analysis. The user can refine the selection until
satisfied.
///
/// Inputs:
/// - `img` (`string`): The name of the image where the RPE area will be selected.
///
/// Outputs:
/// - Adds the RPE selection to the ROI Manager with the name `rpe`.
function rpeAreaSelection(img){
		looper = true;
		setForegroundColor(255, 255, 255);
		setBackgroundColor(0, 0, 0);

		while(looper == true){
				selectWindow(img);
				run("Duplicate...", "duplicate title=RPE");

				Dialog.createNonBlocking("Info");
				Dialog.addMessage("Define RPE area to use in the analysis. \nOK
when ready.");
				Dialog.show();

				run("Clear Outside");
				setAutoThreshold("Li dark");
				run("Convert to Mask");
				run("Morphological Filters", "operation=Closing element=Disk
radius=10");
```

```
				run("Create Selection");
				roiManager("reset");
				roiManager("Add");
				roiManager("select", 0);
				roiManager("Rename", "rpe");
				close("RPE thresholding");

				// Show preview
				selectWindow(img);
				roiManager("show all");

				// Repeat or continue with the selection
				Dialog.createNonBlocking("Continue with the current selection?");
				Dialog.addRadioButtonGroup("Continue or re-do",
newArray("Continue", "Restart RPE-selection") , 1, 2, "Continue");
				Dialog.show();

				buttonSelected = Dialog.getRadioButton();
				if (buttonSelected == "Restart RPE-selection") {
					close("RPE");
					close("RPE-Closing");
					roiManager("delete");
				} else {
					break;
				}
		}
}

/// Executes the h-dome detection for particle detection.
///
/// Inputs:
/// - `fname` (`string`): The filename for logging the output, used only in test mode!
/// - `image` (`string`): The image from which particles will be detected.
/// - `h` (`number`): The `h`-value for dome detection, controlling sensitivity.
/// - `sensitivity` (`number`): The sensitivity threshold for particle detection.
///
/// Outputs:
/// - Prints to the console:
///   - The number of detected particles.
///   - The area of the RPE region.
/// - Updates the ROI Manager with the detected particles.
function run_h_domes(fname, image, h, sensitivity) {
		selectWindow(image);
		mask = getTitle();
		run("8-bit");
		run("Duplicate...", "title=marker");
		run("Subtract...", "value=["+h+"]");

		run("Morphological Reconstruction", "marker=marker mask=["+mask+"] type=[By
Dilation] connectivity=4");
		imageCalculator("Subtract create", mask,"marker-rec");
		rename("hdomes");

		run("Log");
```

```
		run("Morphological Filters", "operation=[White Top Hat] element=Disk radius=4");

		// FOR NORMAL MODE ACTIVATE
		roiManager("select", 0);
		run("Find Maxima..."); // Running this way allows preview
		roiManager("Add");
		roiManager("select", 1);
		roiManager("Rename", "detected particles");

		run("Clear Results");
		run("Set Measurements...", "mean redirect=None decimal=0");
		roiManager("Measure");
		print("---- Found Particles: "+ nResults());

		run("Clear Results");
		roiManager("select", 0);
		run("Set Measurements...", "area redirect=None decimal=0");
		roiManager("Measure");
		print("---- RPE area: " + getResult("Area", 0));
		// FOR NORMAL MODE ACTIVATE ^^^

		// FOR TEST MODE ACTIVATE
		//run("Find Maxima...", "prominence=["+sensitivity+"] output=[Point Selection]");
		//roiManager("Add");
	//roiManager("select", 1);
	//roiManager("Rename", "detected particles");

	//run("Set Measurements...", "centroid redirect=None decimal=3");
	//roiManager("select", 1);
	//roiManager("multi-measure append");

	//for (roi = 0; roi < nResults(); roi++) {
		//setResult("Sensitivity", roi, sensitivity);
		//setResult("h-value", roi, h);
		//setResult("filename", roi, fname);
	// }
	// updateResults();

	//print("---- Found Particles xy: "+ nResults);
	//run("Read and Write Excel", "no_count_column
file=["+user_input[1]+"/evaluation_measurements.xlsx] sheet=["+sheet+"] stack_results");
	//run("Clear Results");
	//roiManager("select", 0);
	// FOR TEST MODE ACTIVATE^^^
}

/// Saves detected ROIs to the output directory.
///
/// Inputs:
/// - `fname` (`string`): The filename used to save the ROIs.
/// - `dir` (`string`): The directory where the ROIs will be saved.
///
/// Outputs:
/// - Saves the ROIs with the name `fname` as a zip file in the `dir` directory.
function saveROIResultsToOutput(fname, dir){
```

```
			roiManager("Select", newArray(0)); // Select RPE and detected particles
			roiManager("Save", dir+"/"+fname+".zip");
}

/// Executes in the test mode by running h-dome detection across various parameter combinations.
///
/// Inputs:
/// - `filename` (`string`): The base filename used for results logging.
/// - `POSimage` (`string`): The image from which particles will be detected.
/// - `hArray` (`array of numbers`): An array of `h`-values for testing different sensitivities.
/// - `sensitivityArray` (`array of numbers`): An array of sensitivity thresholds to test.
///
/// Outputs:
/// - Runs `run_h_domes` multiple times with different combinations of `h` and sensitivity values.
function runTEST(filename, POSimage, hArray, sensitivityArray){
			for (i = 0; i < lengthOf(hArray); i++) {
						print("h-value: "+ hArray[i]);
						for (j = 0; j < lengthOf(sensitivityArray); j++) {
									print("sensitivity: "+ sensitivityArray[j]);
									run_h_domes(filename, POSimage, hArray[i], 
sensitivityArray[j]);
									roiManager("select", 1);
									roiManager("Delete");
									close("hdomes-White Top Hat");
									close("hdomes");
									close("marker-rec");
									close("marker");
						}
			}
			print("DONE, all saved");
}

// Main Macro Execution

testMode = false; // Set to true for testing
sensitivity = newArray(5, 10, 20, 40, 60); // Sensitivity thresholds for tests
hvalues = newArray(5, 15, 50, 100, 300); // h-dome values for tests
CHANNELS = newArray("1", "2", "3", "4"); // Available channels

user_input = startup();
filelist = getFileList(user_input[0]);

for (i = 0; i < lengthOf(filelist); i++) {
			if (endsWith(filelist[i], ".nd2")) {
						print("Processing: "+filelist[i]);
						run("Bio-Formats Importer", "open=[" + user_input[0] + filelist[i] + 
"] autoscale color_mode=Default rois_import=[ROI manager] view=Hyperstack 
stack_order=XYCZT");
						filename = File.getNameWithoutExtension(getTitle());
						getDimensions(width, height, channels, slices, frames);
						run("Z Project...", "projection=[Max Intensity]");
						run("Gaussian Blur...", "sigma=1 stack");
						Stack.setDisplayMode("composite");

						analysis_channels = channelCheck();
```

```
                rpe = analysis_channels[0];
                particle = analysis_channels[1];

                rpeAreaSelection(rpe);

                if(testMode == true){
                        runTEST(File.getNameWithoutExtension(filelist[i]),
particle, hvalues, sensitivity);

        saveROIResultsToOutput(File.getNameWithoutExtension(filelist[i]), user_input[1]);
                } else {
                        run_h_domes("", particle, user_input[2], 50);
                        selectWindow(particle);
                        roiManager("select", 1);
                        waitForUser("OK to save results");
                        saveROIResultsToOutput(filename, user_input[1]);
                        close("*");
                }
        }
}
```

Text S1

B) Parameters and their definitions and mathematical equations needed for the evaluation of the performance of the analysis tool's peak detection algorithm

**Parameter**	**Definition**	**Equation**
Precision	The proportion of detected peaks that were correct	$Precision = \frac{TP}{(TP + FP)}$
Ground truth	The manually annotated local intensity maxima	
Sensitivity (Recall)	The proportion of ground truth peaks that were successfully detected	$Sensitivity = \frac{TP}{(TP + FN)}$
F1-score (F1)	The harmonic mean of precision and sensitivity, providing a single measure of performance	$F1 = \frac{2x(Precision\ x\ sensitivity)}{(Precision + sensitivity)}$
False Positive Rate (FPR)	The proportion of negative ground truth peaks incorrectly classified as positive	$FPR = \frac{FP}{FP + TP}$
True positive (TP)	A detected peak that was within three pixels of a ground truth peak	
False positive (FP)	A detected peak that could not be matched to any ground truth peak	
False Negative (FN)	A ground truth peak that was not matched to any detected peak	

Text S1

C)

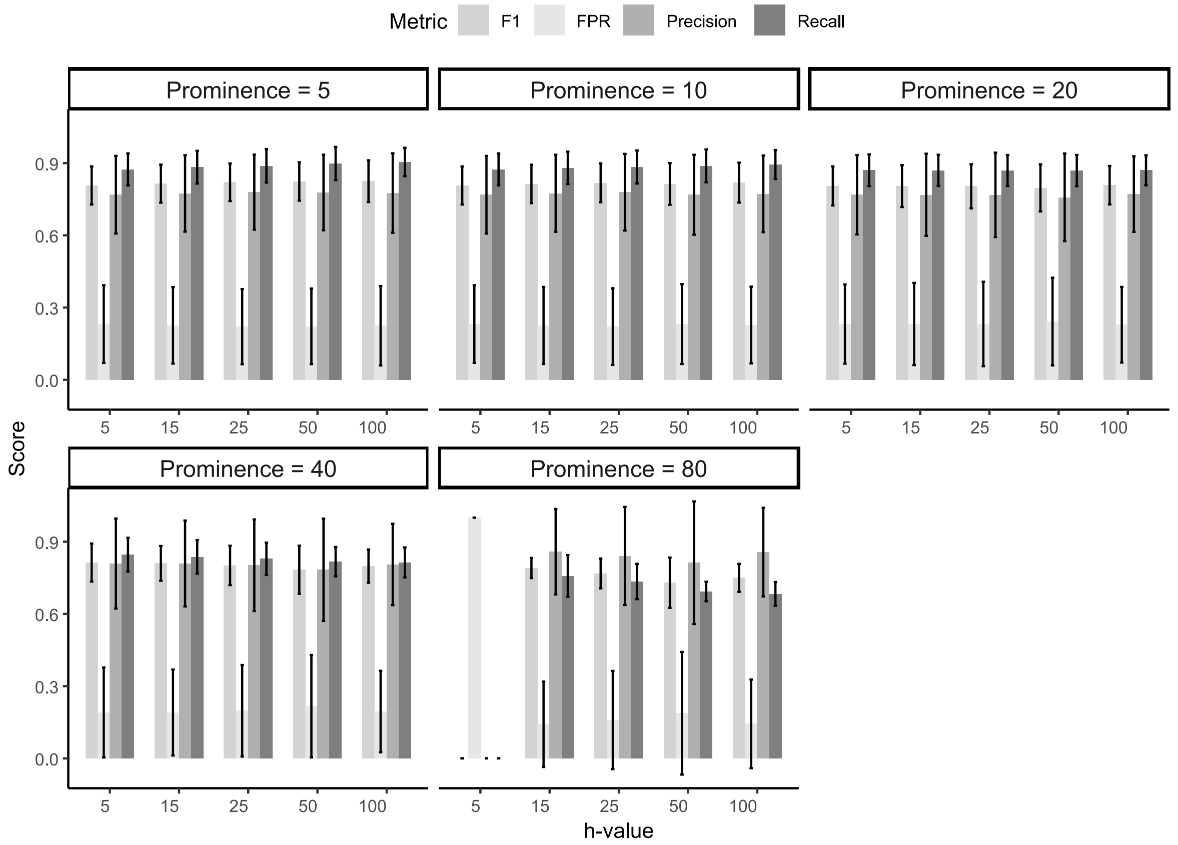

Text S1. Comparison of the performance of the analysis tool's peak detection algorithm using different combinations of five h-dome values and five prominence thresholds across three randomly selected sample images. *The plots show the calculated score values for precision, Recall (sensitivity), F1-score (F1), and false positive rate (FPR) on the Y-axis for each h-dome-prominence threshold combination, allowing for an evaluation of the algorithm's overall detection reliability. The tested h-values are on the X-axis. The results suggest that the h-value has minimal impact on the algorithm's performance to detect True positive (TP) intensity peaks. Moreover, the data indicates that the performance remains nearly identical with the prominence threshold values set between 5 and 20 with each h-values. However, around prominence threshold value 80, and especially in combination with low h-values, the algorithm's performance seems to decline. This is likely due to over-filtering. For the final analysis tool, h-value was set to 15 and the prominence threshold values is defined by the experimenter specifically for each image sample.*
