# Supplementary material for: Phagocytosed Photoreceptor Outer Segment Particles Within the Retinal Pigment Epithelium Show Diurnal Rhythmicity and Variation Between Cone Subtypes in Larval Zebrafish
Source: FASEB J. 2025 Jul 24;39(14):e70853. doi: 10.1096/fj.202500211R (PMC12288107; doi:10.1096/fj.202500211R)
Supplement: Supplementary file 1 — Appendix S1. [file FSB2-39-e70853-s001.zip › fsb270853-sup0003-Figure S1.pdf]

Supplemental material
Figure S1

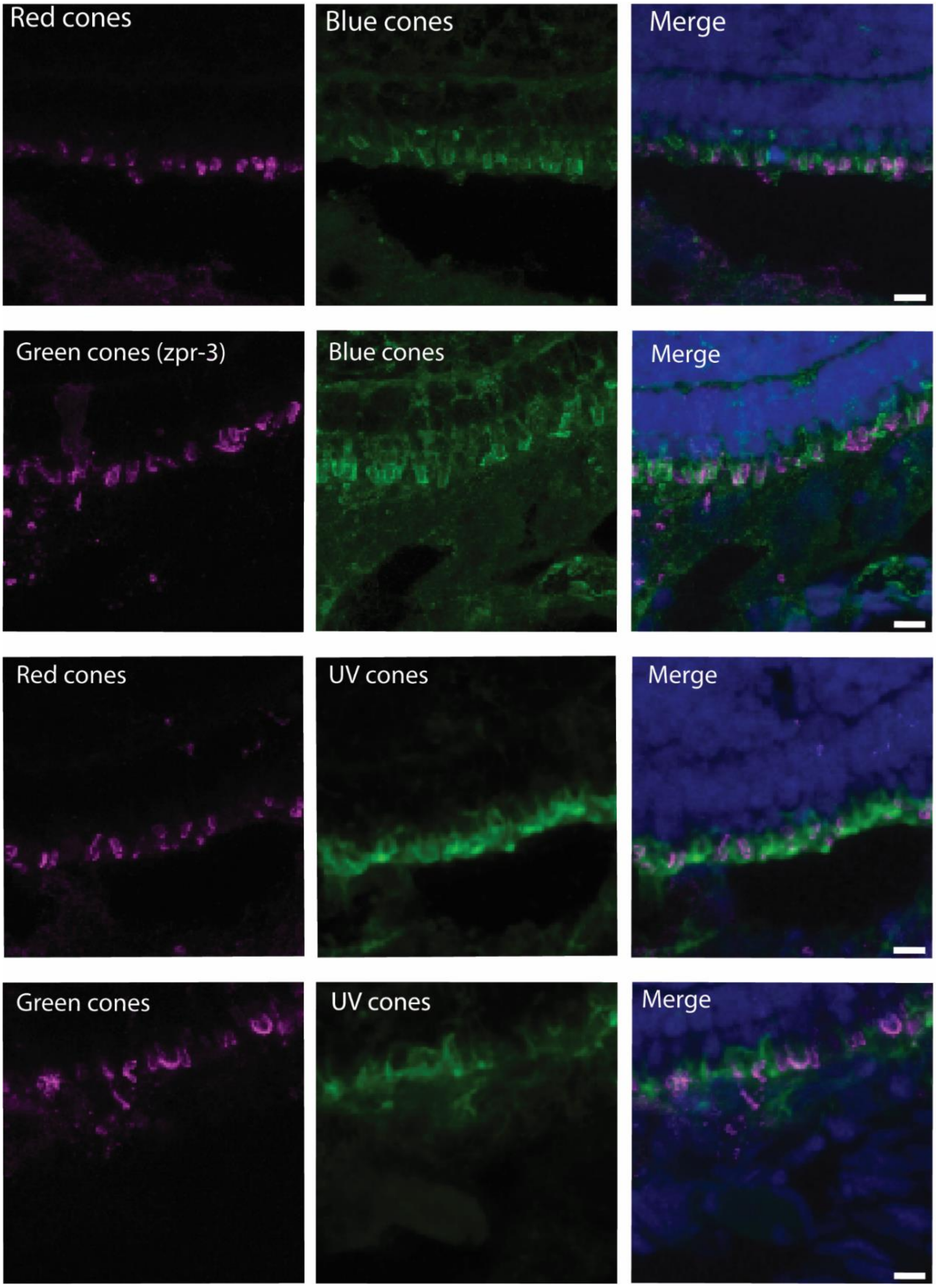

Fig S1. Immunofluorescence labelling of 7 *dpf* old zebrafish cone subtypes. *Different antibody combinations (magenta and green) were used to label simultaneously two different cone subtypes together with DAPI (Blue). The confocal images of all the combinations show that the OSs of different cone subtypes appear in the same layer in zebrafish at 7 dpf. Scale bars 5 µm. OSs: outer segments.*
