# Supplementary material for: Phagocytosed Photoreceptor Outer Segment Particles Within the Retinal Pigment Epithelium Show Diurnal Rhythmicity and Variation Between Cone Subtypes in Larval Zebrafish
Source: FASEB J. 2025 Jul 24;39(14):e70853. doi: 10.1096/fj.202500211R (PMC12288107; doi:10.1096/fj.202500211R)
Supplement: Supplementary file 1 — Appendix S1. [file FSB2-39-e70853-s001.zip › fsb270853-sup0004-Figure S2.pdf]

Supplemental material

Figure S2

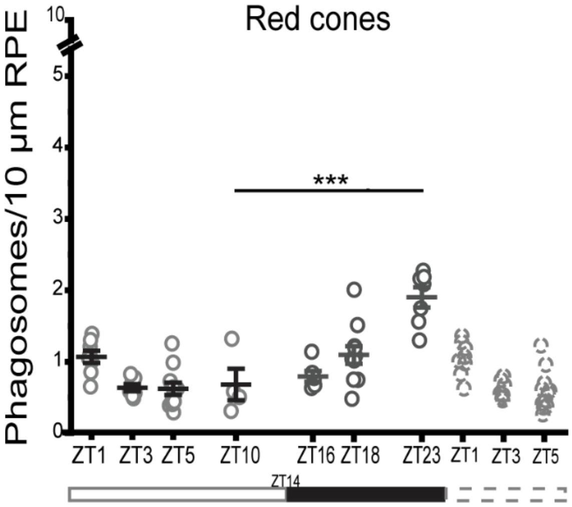

Time	Red cones in LD
ZT1	1.07
ZT3	0.63
ZT5	0.62
ZT10	0.69
ZT16	0.82
ZT18	1.12
ZT23	1.93
Average of total OS phagosome count over 24 hours	0.98

Fig S2. The numbers of phagosomes from red cone OSs in the RPE over the 24 h period in normal light-dark cycle. *The scatter plot shows the number of phagosomes from red cone OSs per 10 µm of RPE at each studied time point. Data is represented as individual samples (circles) together with the mean line ± SEM. One-way ANOVA analysis was used to show statistically significant differences in OS phagosome numbers over the 24 hours. Subsequent Bonferroni's post hoc test revealed significant increase in OS phagosomes at ZT23 (1.93 phagosomes/10 µm RPE) compared to the baseline at ZT10 (0.69 phagosomes/10 µm RPE) (***p<.001). The white bar and the black bar represent the light and dark periods of the day, respectively. The dashed bar shows again the three first time points of the day. The table shows averages of the phagosomes from the red cone OSs per 10 µm of RPE at each time point under normal light-dark cycle. Phagosomes were quantified from the length of the entire RPE tissue in each whole eye section. N≥5 sections at each time point, each section represents one eye. RPE: Retinal pigment epithelium, ZT: Zeitgeber time, White bar under the graph: light period of the day, Black bar under the graph: dark period of the day, OS: outer segment.*
