# Supplementary material for: Phagocytosed Photoreceptor Outer Segment Particles Within the Retinal Pigment Epithelium Show Diurnal Rhythmicity and Variation Between Cone Subtypes in Larval Zebrafish
Source: FASEB J. 2025 Jul 24;39(14):e70853. doi: 10.1096/fj.202500211R (PMC12288107; doi:10.1096/fj.202500211R)
Supplement: Supplementary file 1 — Appendix S1. [file FSB2-39-e70853-s001.zip › fsb270853-sup0005-Figure S3.pdf]

<u>Supplemental material</u>

Figure S3

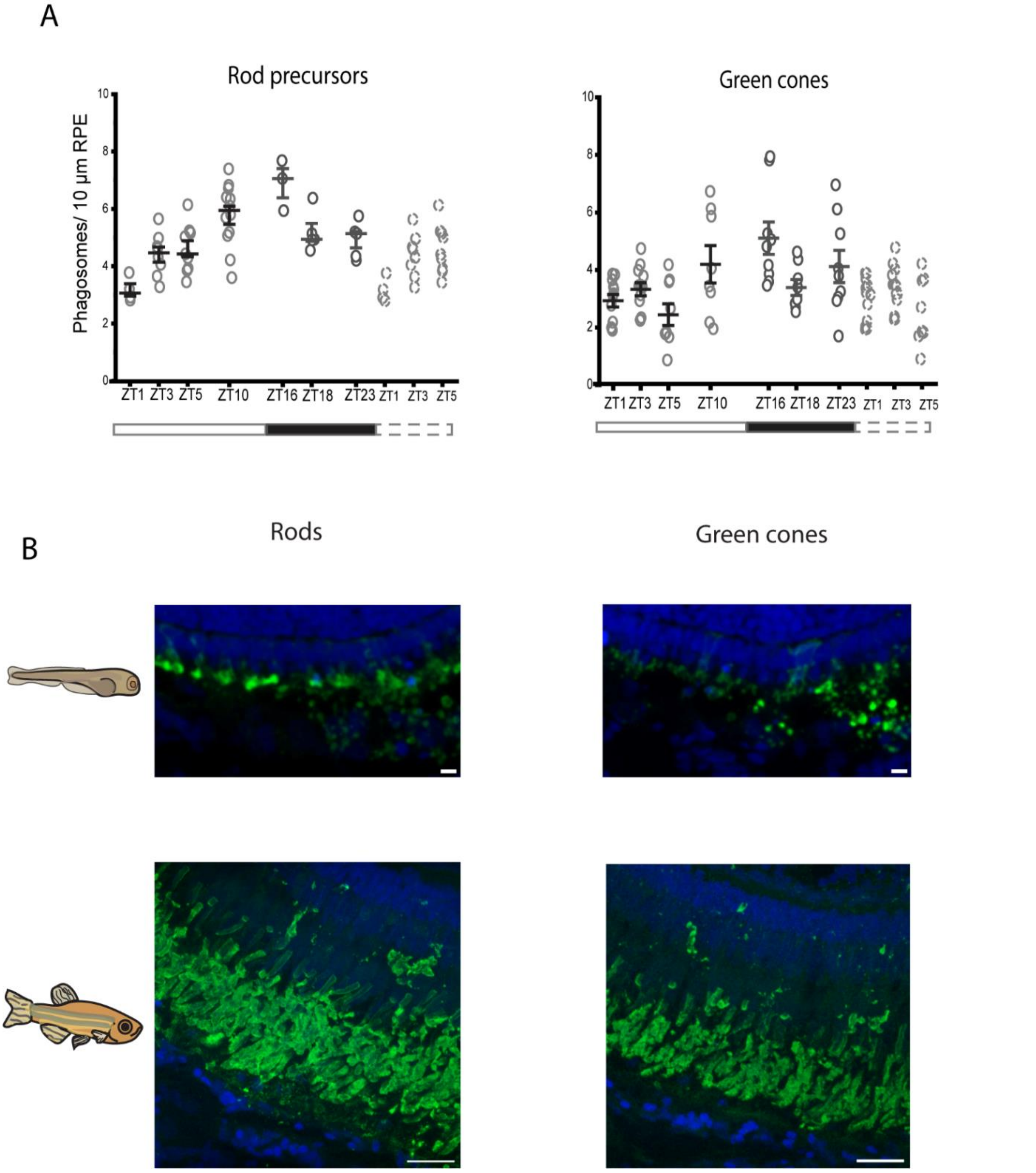

*Fig S3. **Quantitative and image data of rods and green cones** A) Graphs show the number of phagosomes from rod precursor OSs and green cone OSs per 10 µm of RPE at each studied time point. Data is represented as individual samples (circles) together with the mean line ± SEM. Rhythmic variation in the numbers of phagosomes originating from rod precursor OSs shows similar trend to that of green cone OSs. The white bar and the black bar represent the light and dark periods of the day, respectively. The dashed bar shows again the three first time points of the day. B) Immunofluorescent labelling of 7 dpf larval (wild-type) and adult (Fli-eGFP strain) zebrafish cryosections using DAPI stain (Blue) together with either rod opsin antibody (O4886) or zpr-3 to visualize rods (green) and green*

cones (green), respectively. In adult sections (lower row), these antibodies seem to label both rod and cone layers. Scalebar in larval sections: 5 µm and in adult sections: 20 µm.
