# Supplementary material for: Phagocytosed Photoreceptor Outer Segment Particles Within the Retinal Pigment Epithelium Show Diurnal Rhythmicity and Variation Between Cone Subtypes in Larval Zebrafish
Source: FASEB J. 2025 Jul 24;39(14):e70853. doi: 10.1096/fj.202500211R (PMC12288107; doi:10.1096/fj.202500211R)
Supplement: Supplementary file 1 — Appendix S1. [file FSB2-39-e70853-s001.zip › fsb270853-sup0006-Figure S4.pdf]

Supplemental material

Figure S4

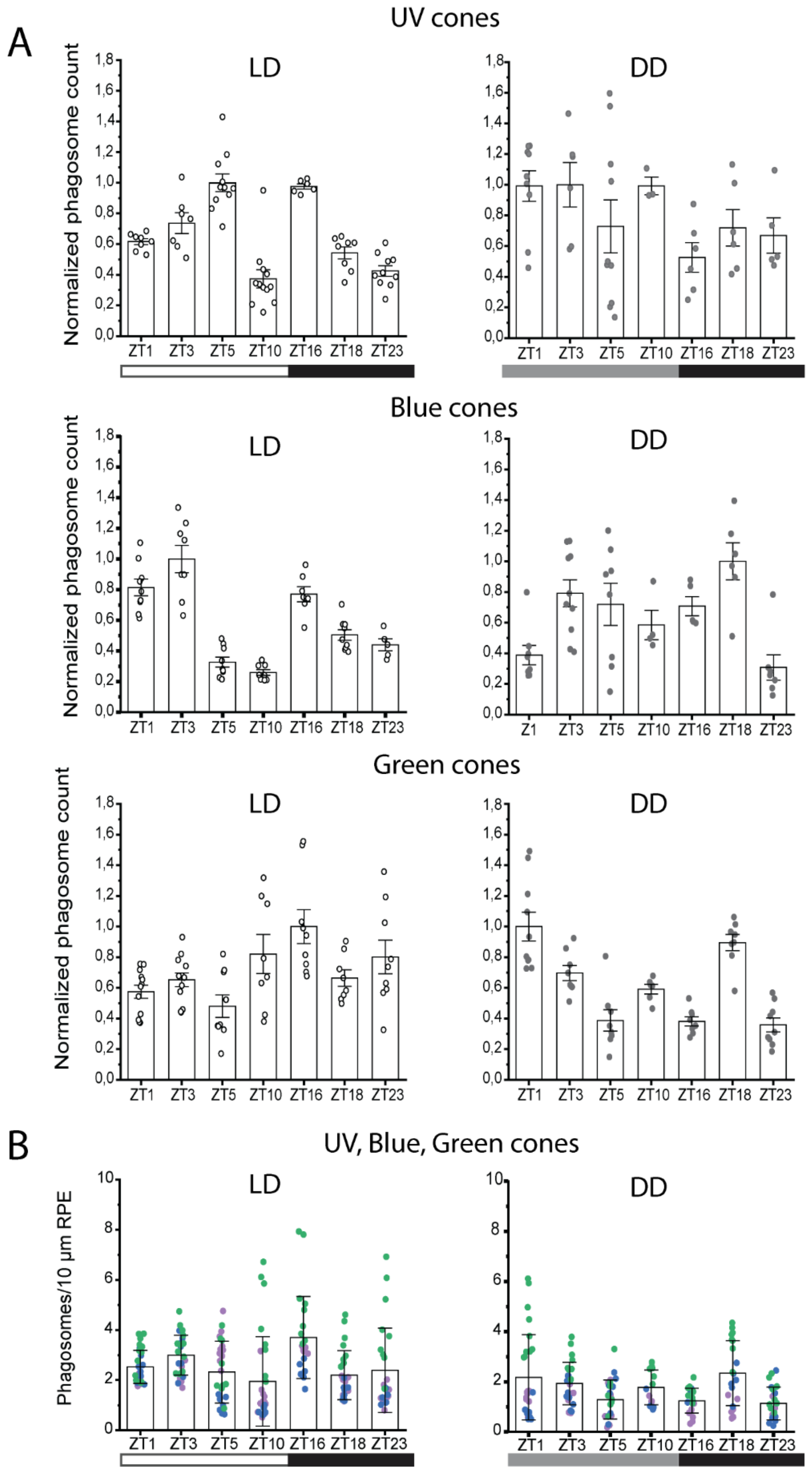

Fig. S4. The number of phagosomes from the UV and blue cone OSs show two daily peaks in LD, but not in DD in larval zebrafish. *A) Quantified phagosome numbers for UV, blue and green cone OSs were normalized to the level of the highest mean of OS phagosomes (phagosomes/10 µm of RPE) over the studied time points individually for each cone subtype in LD and DD conditions. The plots show each individual sample as a circle and the bar represents the mean ± SEM. B) Non-normalized phagosome counts for UV, blue and green cone OSs at studied time points in LD and DD. The plots show individual samples as circles with different colors as follows: Purple: UV cones, blue: blue cones and green: green cones. The bar represents the mean ± SD. OSs: Outer segments, LD: normal light cycle, DD: constant darkness*
